# *Meioc* maintains an extended meiotic prophase I in mice

**DOI:** 10.1371/journal.pgen.1006704

**Published:** 2017-04-05

**Authors:** Y. Q. Shirleen Soh, Maria M. Mikedis, Mina Kojima, Alexander K. Godfrey, Dirk G. de Rooij, David C. Page

**Affiliations:** 1 Whitehead Institute, Cambridge, MA, United States of America; 2 Department of Biology, Massachusetts Institute of Technology, Cambridge, MA, United States of America; 3 Howard Hughes Medical Institute, Whitehead Institute, Cambridge, MA, United States of America; Cornell University, UNITED STATES

## Abstract

The meiosis-specific chromosomal events of homolog pairing, synapsis, and recombination occur over an extended meiotic prophase I that is many times longer than prophase of mitosis. Here we show that, in mice, maintenance of an extended meiotic prophase I requires the gene *Meioc*, a germ-cell specific factor conserved in most metazoans. In mice, *Meioc* is expressed in male and female germ cells upon initiation of and throughout meiotic prophase I. Mouse germ cells lacking *Meioc* initiate meiosis: they undergo pre-meiotic DNA replication, they express proteins involved in synapsis and recombination, and a subset of cells progress as far as the zygotene stage of prophase I. However, cells in early meiotic prophase—as early as the preleptotene stage—proceed to condense their chromosomes and assemble a spindle, as if having progressed to metaphase. *Meioc*-deficient spermatocytes that have initiated synapsis mis-express CYCLIN A2, which is normally expressed in mitotic spermatogonia, suggesting a failure to properly transition to a meiotic cell cycle program. MEIOC interacts with YTHDC2, and the two proteins pull-down an overlapping set of mitosis-associated transcripts. We conclude that when the meiotic chromosomal program is initiated, *Meioc* is simultaneously induced so as to extend meiotic prophase. Specifically, MEIOC, together with YTHDC2, promotes a meiotic (as opposed to mitotic) cell cycle program via post-transcriptional control of their target transcripts.

## Author summary

Meiosis is the specialized cell division that halves the genetic content of germ cells to produce haploid gametes. This reductive division is preceded by a preparative phase of the cell cycle, meiotic prophase I, during which several meiosis-specific chromosomal events occur. Across sexually reproducing organisms, prophase of meiosis I is dramatically longer than mitotic prophase. However, it was not known in mammals how and why meiotic prophase I is extended. We have identified a mouse mutant in which this extended prophase I is disrupted: germ cells lacking *Meioc* initiate meiosis, but prematurely proceed to metaphase. Mutant male meiotic germ cells mis-express a cell cycle regulator that is normally expressed in mitotic male germ cells, suggesting that *Meioc* is required for germ cells to properly transition to a meiotic cell cycle program. Biochemical analyses of proteins and transcripts that associate with MEIOC protein suggest that MEIOC may promote the transition from a mitotic to meiotic cell cycle program by post-transcriptionally regulating target transcripts. Our studies indicate that in mammals, as in other sexually reproducing organisms, meiotic prophase I must be extended to allow time for meiotic chromosomal events to reach completion.

## Introduction

Meiosis is a specialized cell division program that results in the halving of parental genetic material and the production of haploid gametes. This reductive division depends on a series of chromosomal events that occur specifically during meiotic but not mitotic prophase, including the loading of meiosis-specific cohesins on sister chromatids, alignment and synapsis of homologous chromosomes, and generation of covalent linkages between homologs via recombination. These meiotic chromosomal events occur during meiotic prophase I, which takes much longer than mitotic prophase. In yeast, it has been shown that completion of these chromosomal events requires the extended prophase I: yeast meiotic prophase I lasts 3.5 hours, compared to 15 minutes for mitotic prophase [1], and premature exit from prophase I results in recombination defects and chromosome missegregation [2].

Mammals similarly have an extended prophase I. In female mice, ovarian germ cells initiate meiosis around embryonic day 13.5 (E13.5), and arrest in the penultimate stage of prophase, diplotene, around the time of birth, one week after meiotic initiation [3,4]. In male mice, cohorts of testicular germ cells initiate meiosis continuously throughout post-pubertal life, each cohort taking two weeks from initiation to completion of meiotic prophase I [5]. In contrast, the typical mitotic prophase in mammalian cells lasts only minutes [6,7].

No mechanism for extension of meiotic prophase has yet been recognized in mammals. In other organisms, the extension of meiotic prophase is accomplished by meiosis-specific modifications of the cell cycle. In yeast, exit from meiotic prophase I is postponed via the suppression of mitotic cell cycle regulators by a meiosis-specific form of the anaphase-promoting complex [2]. In worm and fly, exit from meiotic prophase I is also actively suppressed by meiosis-specific factors via translational repression of, respectively, cyclins E and A [8,9].

Since meiotic initiation in both male and female mice is governed by the retinoic acid-induced gene *Stra8* [10,11], STRA8 activity might be at least indirectly related to prolonging prophase. Ovarian and testicular germ cells express *Stra8* shortly before entering meiotic prophase I [12,13], and *Stra8* is required for the chromosomal events of meiotic prophase I, including cohesion, synapsis, and recombination [14,15]. Consistent with a pivotal role in meiotic initiation, most genes involved in meiotic prophase I depend on *Stra8* for their expression. However, *Stra8* is only transiently expressed at the time of meiotic initiation, and therefore is unlikely to be the factor responsible for maintaining meiotic prophase I. We previously identified a subset of early meiotic genes that are expressed independently or partially independently of *Stra8*, and are induced concurrently or shortly after *Stra8* [16,17]. This subset of partially *Stra8*-independent early meiotic genes includes cohesins and synaptonemal complex proteins with known meiotic functions, and also *Meioc*, an uncharacterized gene formerly named *Gm1564*.

We examined MEIOC expression and find that it is expressed throughout meiotic prophase I in both testicular and ovarian germ cells; this expression profile suggests that its function begins early and persists throughout meiotic prophase I in both sexes. We examined mice deficient for *Meioc*, and found that *Meioc*-deficient germ cells can initiate but do not complete meiotic prophase I. Instead, germ cells that have initiated meiosis proceed prematurely to an aberrant metaphase. *Meioc*-deficient germ cells that have initiated meiosis mis-express CCNA2, which is typically expressed in mitotic spermatogonia, suggesting a failure to properly transition to a meiotic cell cycle program. We propose that *Meioc* functions continuously throughout meiotic prophase I to prevent premature exit from prophase I, likely by promoting a meiotic (as opposed to mitotic) cell cycle program. Further, MEIOC interacts with an RNA helicase, YTHDC2, and binds a common set of germ cell transcripts, suggesting that MEIOC and YTHDC2 partner to regulate these transcripts.

Our observations that *Meioc*-deficient germ cells fail to complete meiotic prophase I and instead produce numerous abnormal metaphases are concordant with a recent study [18]. However, whereas Abby and colleagues propose that this phenotype results from arrested meiotic progression, our molecular analyses of cyclin expression and MEIOC-bound transcripts lead us to an alternate interpretation of the phenotype–that the precocious metaphases observed are a result of cell cycle mis-regulation. We propose a model for meiotic prophase I as comprised of multiple subprograms: these include the chromosomal program, whereby chromosomes synapse and undergo recombination, and a meiosis-specific cell cycle program, whereby cells are maintained in an extended prophase I to allow completion of the chromosomal program.

## Results

### *Meioc* is a conserved, germ cell-specific gene expressed during male and female meiotic prophase I

We had previously identified *Meioc* (*Gm1564*) as one of the earliest and most strongly induced transcripts upon meiotic initiation in the female germline [17]. In the study presented here, we identified full-length *Meioc* homologs in almost all vertebrate genomes examined. Furthermore, we found that *Meioc*’s conserved C-terminal domain, PF15189 (previously DUF4582), is present approximately once per genome in almost all metazoan genomes examined (S1 Fig). We were unable to identify orthologs of *Meioc* or matches to PF15189 in Diptera, including *Drosophila melanogaster*, which hints at *Meioc* being replaced functionally by alternate proteins or pathways in this lineage. Next, we examined *Meioc* expression in adult tissue panels from human, mouse, rat, and chicken, and found its expression to be highly testis-specific (S2 Fig). Thus, *Meioc* is a highly conserved gene whose expression pattern across diverse species is consistent with a role in meiosis.

**Fig 1:**
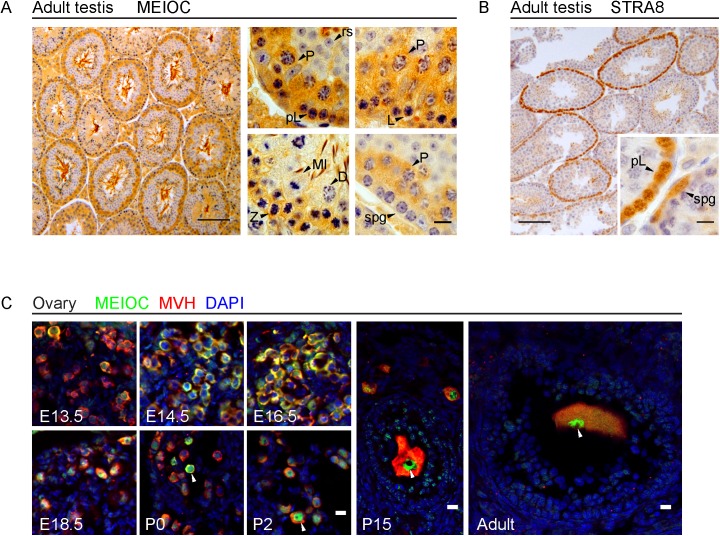
MEIOC is expressed throughout most of meiotic prophase I in the male and female germline. (A) Immunohistochemistry for MEIOC (brown) in adult testis, with hematoxylin counterstaining to enable identification of germ cell types by nuclear morphology [57]. Low magnification image shows MEIOC staining in the majority of meiotic cell populations. Background staining was also observed in mature sperm in center of the tubule. High magnification images show that MEIOC was detected in meiotic germ cells at preleptotene (pL), leptotene (L), zygotene (Z), and pachytene (P) stages; it was not detected in meiotic germ cells at diplotene (D) stage, in cells undergoing meiotic metaphase (MI), or in postmeiotic round spermatids (rs). Low magnification scale bar = 100 μm, high magnification scale bar = 10 μm. (B) Immunohistochemistry for STRA8 (brown) in adult testis, counterstained with hematoxylin. In contrast to MEIOC, STRA8 expression is limited to germ cells initiating meiosis (preleptotene and leptotene stages) as well as differentiating spermatogonia. Scale bar = 100 μm. (C) Immunofluorescence staining for MEIOC in ovary at E13.5, E14.5, E16.5, E18.5, P0, P2, P15, and adult (>8 weeks). Mouse Vasa Homolog (MVH) costaining identifies germ cells [58]. Nuclei stained by DAPI. MEIOC is detected in germ cells at all stages. From E13.5 to E18.5, corresponding to premeiotic to late pachytene stages, MEIOC is detected predominantly in cytoplasm. Towards the end of this period, MEIOC is detected in nucleus (arrowheads), and continues to be expressed in nuclei of germ cells at postnatal timepoints. Scale bar = 10 μm.

To determine the precise cell types in which MEIOC is expressed, we generated a rabbit polyclonal antibody to a C-terminal fragment of MEIOC, which we verified to be specific using subsequently generated *Meioc*-deficient mice (S3 Fig). Immunohistochemistry for MEIOC on adult testis sections showed that MEIOC is expressed in spermatocytes, beginning in preleptotene and extending through most stages of meiotic prophase I, including leptotene, zygotene, and pachytene, but not during diplotene and diakinesis (the final stages of meiotic prophase I) or meiotic metaphase I (Fig 1A). MEIOC is absent in spermatogonia, in post-meiotic spermatids, and in somatic cells. Subcellular localization of MEIOC during early to mid-prophase I was predominantly cytoplasmic, but by late pachytene a fraction of MEIOC was nuclear. The prolonged expression of MEIOC contrasts starkly with that of STRA8, which is similarly induced in preleptotene cells, but then rapidly down regulated once cells enter leptotene (Fig 1B).

To determine if MEIOC is expressed at similar stages of meiotic prophase in the female, we immunostained for MEIOC on fetal ovary sections (Fig 1C). MEIOC was detected by E13.5, when germ cells are preparing to enter meiosis, and persists through leptotene, zygotene, pachytene stages of meiotic prophase, and dictyate arrest in the adult. In females, MEIOC is initially predominantly cytoplasmic, but becomes predominantly nuclear postnatally. Thus, MEIOC is similarly expressed in both sexes: beginning at meiotic initiation, and persisting through most of meiotic prophase in the male, and through to dictyate arrest in the female. To our knowledge, this combination of germ-cell-specific expression throughout most of meiotic prophase and predominantly cytoplasmic localization is unique to MEIOC. Our characterization of MEIOC expression broadly agrees with results obtained using antibodies generated against full-length MEIOC [18], with a few exceptions: Abby and colleagues reported an exclusively cytoplasmic localization, whereas we observed MEIOC attaining nuclear localization towards the end of meiotic prophase.

### *Meioc*-deficient mice are infertile

To explore the role of *Meioc* in germ cell differentiation and meiotic prophase, we generated *Meioc*-deficient mice using a targeting vector generated by the Knockout Mouse Project (KOMP) (S4 Fig). Results reported here were performed in mice 5 to 7 generations backcrossed to the C57BL/6 background unless otherwise stated. *Meioc*-deficient mice (*Meioc* -/-) had markedly smaller ovaries and testes than did wild-type control (*Meioc* +/-, and *Meioc* +/+) mice (S5A Fig), and they were infertile.

**Fig 2:**
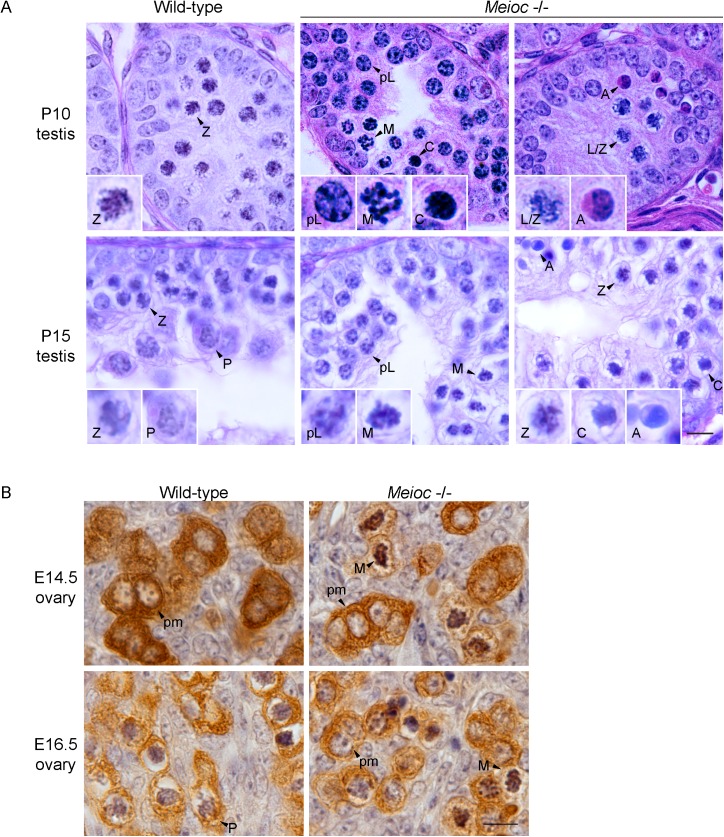
*Meioc*-deficient testicular and ovarian germ cells fail to progress through meiotic prophase, and instead exhibit metaphase-like chromosome condensation. (A) Hematoxylin and eosin stain of wild-type and *Meioc* -/- testes at P10 and P15. Germ cell types are identified by nuclear morphology and position within tubules [57]. In wild-type P10 testis, germ cells have advanced to zygotene (Z) stage of meiotic prophase. In wild-type P15 testis, germ cells have advanced to an epithelial stage showing already two generations of spermatocytes: zygotene (Z) and pachytene (P) stages of meiotic prophase. In *Meioc* -/- P10 and P15 testes, germ cells in center of lumen and adjacent to preleptotene-stage cells exhibited metaphase-like chromosome condensation (M). Some germ cells also progress through leptotene (L) to late leptotene/early zygotene (L/Z). In addition, cells with condensed (C) and apoptotic nuclei (A) are observed. In *Meioc* -/- P15 testes, we observed no pachytene-stage cells. Scale bar = 10 μm. (B) Immunohistochemistry for MVH in fetal ovaries, counterstained with hematoxylin. In wild-type E14.5 ovary, germ cells exhibited a premeiotic morphology (pm). In *Meioc* -/- E14.5 ovary, some germ cells also exhibited a premeiotic morphology (pm), but other germ cells exhibited metaphase-like chromosome condensation (M). In wild-type E16.5 ovary, germ cells had progressed to late zygotene/early pachytene (P) stages of meiotic prophase. This was not observed In *Meioc* -/- E16.5 ovary; instead, germ cells either retained a premeiotic morphology (pm), or exhibited metaphase-like chromosome condensation (M). Scale bar = 10 μm.

*Meioc*-deficient adult testes completely lacked post-meiotic germ cells (S5B and S5C Fig) and were dramatically depleted of cells in meiotic prophase I compared to littermate controls. To study progression through meiotic prophase I in a synchronous setting, we examined testes at 10 and 15 days after birth (P10 and P15) to follow the meiotic development of the first cohort of spermatogenic cells (Fig 2A). By P10 in wild-type testes, spermatogenic cells have initiated meiosis and progressed from preleptotene to the leptotene and zygotene stages of meiotic prophase. By P15, the most advanced spermatogenic cells have transitioned through zygotene and progressed to the pachytene stage. No later stages of meiosis, namely diplotene and meiotic metaphases, are observed at P10 and P15. In *Meioc*-deficient mutants, P10 and P15 testes contained cells with chromosomes condensed like those observed during metaphase. Meiotic metaphases are not expected until P20, and were not observed in our control P10 and P15 wild-type testes. *Meioc*-deficient testes also contained cells with abnormal condensed nuclei, and apoptotic cells. Mutant testes also contained leptotene and zygotene-stage spermatocytes, but were devoid of pachytene spermatocytes. TUNEL-positive cells were rare in wild-type adult testes but were abundant in *Meioc*-deficient adult testes, specifically in cells with condensed or apoptotic nuclei (S6 Fig). TUNEL staining was not observed in preleptotene, leptotene, zygotene-like, or metaphase-like cells of *Meioc*-deficient testes.

To determine if similar defects occur in females, we examined *Meioc*-deficient and wild-type ovaries. In contrast to wild-type adult ovaries, which contain follicles at various stages of maturation, adult ovaries of *Meioc*-deficient females contain no oocytes or follicles (S5D Fig). In wild-type fetal ovaries, germ cells progress from a premeiotic stage at E14.5 to zygotene or pachytene stages by E16.5 (Fig 2B). In *Meioc*-deficient ovaries, metaphase-like cells were observed as early as E14.5, and persist to E16.5. Most remaining germ cells exhibited premeiotic morphology, and few reached the leptotene or zygotene stages of prophase, even by E16.5. An independent *Meioc* knockout mouse line generated using the same KOMP vector on a mixed genetic background (C57BL/6 crossed to NMRI) exhibited similar histological phenotypes [18].

To pinpoint the timing of the primary defect in *Meioc*-deficient germ cells, we examined the time and stage at which aberrant metaphase-like cells first arise. In the testis, they are found adjacent to preleptotene, leptotene, and zygotene spermatocytes (Figs 2A and S7). The occurrence of metaphase-like cells adjacent to preleptotene cells in stage VIII tubules suggests that metaphase-like cells first arise shortly after the preleptotene stage, before recombination and synapsis would normally occur. Some germ cells proceed somewhat further, to the leptotene or zygotene stage, possibly because the primary defect that causes premature metaphase is not completely penetrant at the preleptotene stage. In the ovary, metaphase-like cells arise as early as E14.5, when most wild-type germ cells are still in the pre-meiotic stage. Thus, in both sexes, the primary defect that causes premature metaphase occurs shortly after the decision to initiate meiosis, and prior to meiotic chromosomal events such as recombination and synapsis.

### *Meioc*-deficient testicular and ovarian germ cells exhibit molecular markers of meiotic initiation and early meiotic prophase

To confirm that *Meioc-*deficient germ cells have initiated meiotic prophase I, we examined *Meioc*-deficient testes and ovaries for molecular markers of meiotic initiation and early meiotic prophase I.

**Fig 3:**
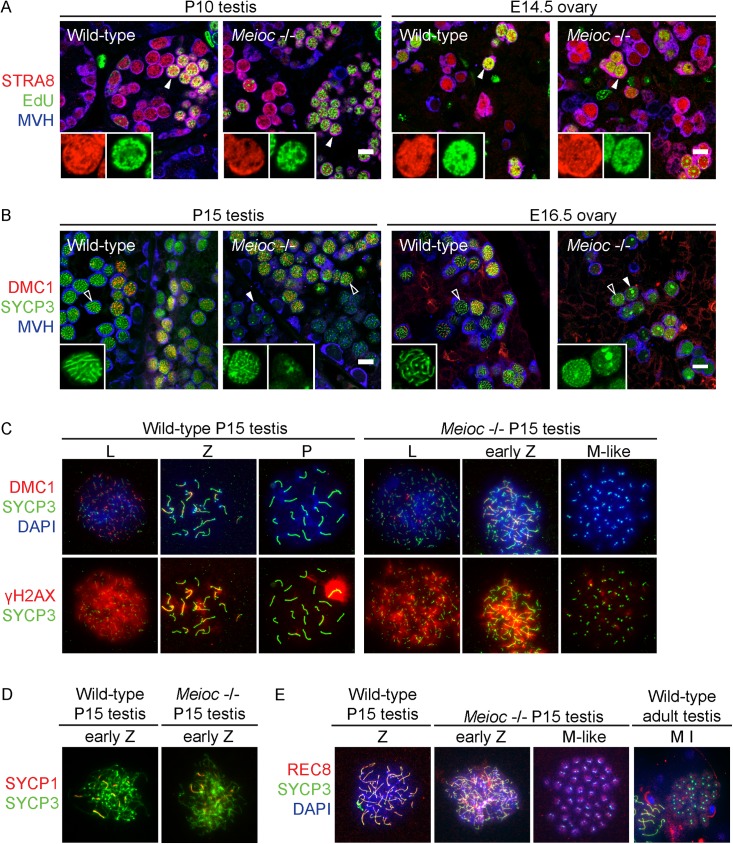
*Meioc*-deficient testicular and ovarian germ cells express molecular markers of meiotic prophase, but do not progress past early zygotene. (A) Immunofluorescence staining for STRA8, MVH, and EdU incorporation, in wild-type and *Meioc* -/- P10 testis and E14.5 ovary sections. Insets: Higher magnification, STRA8 and EdU staining. In wild-type P10 testis, STRA8 expression is seen in a subset of MVH+ germ cells (arrowhead and inset), indicative of these cells initiating meiosis. STRA8+ germ cells are also observed in the *Meioc* -/- P10 testis. In wild-type and *Meioc* -/- E14.5 ovaries, STRA8 expression is visible in most MVH+ germ cells (arrowhead and inset), indicative of germ cells synchronously initiating meiosis. In wild-type and *Meioc* -/- ovaries of both sexes, some STRA8+ cells are also EdU+ (arrowhead and inset), reflecting premeiotic DNA synthesis. Scale bar = 10 μm. (B) Immunofluorescence staining for DMC1, SYCP3, and MVH, in wild-type and *Meioc* -/- P15 testis and E16.5 ovary sections. Insets: Higher magnification, SYCP3 staining. In wild-type P15 testis, we expected to observe germ cells in leptotene, zygotene, and pachytene (empty arrowhead and inset) stages of meiotic prophase. DMC1 expression and SYCP3 localization along the chromosomes is consistent with these stages. In *Meioc* -/- P15 testis, expression of both DMC1 and SYCP3 is seen, but the pattern of SYCP3 localization does not progress beyond what is typical of early zygotene, and is often accompanied by SYCP3 aggregates (empty arrowhead and inset). Additionally, some germ cells contain only SYCP3 aggregates (filled arrowhead and inset). In wild-type E16.5 ovary, we expected most germ cells to be in pachytene of meiotic prophase. DMC1 expression and SYCP3 localization along the chromosomes are consistent with pachytene stage (empty arrowhead and inset). In *Meioc* -/- E16.5 ovary, DMC1 expression and SYCP3 expression are also observed, but the pattern of SYCP3 localization does not progress beyond what is typical of early zygotene, and is often accompanied by SYCP3 aggregates (empty arrowhead and inset). Some germ cells contain only SYCP3 aggregates (filled arrowhead and inset). Scale bar = 10 μm. (C) Immunofluorescence staining for DMC1, γH2AX, and SYCP3 in chromosome spreads of wild-type and *Meioc* -/- germ cells from P15 testis. DNA stained by DAPI. In wild-type germ cells, we observed DMC1, γH2AX, and SYCP3 localization consistent with leptotene, zygotene, and pachytene stages of meiotic prophase. In some *Meioc* -/- germ cells, we observed DMC1, γH2AX, and SYCP3 staining indicative of leptotene and early zygotene stages. In metaphase-like cells, we observed SYCP3 localization at the ends of chromosomes, likely at centromeres. (D) Immunofluorescence staining for SYCP3 and SYCP1 in chromosome spreads of wild-type and *Meioc* -/- zygotene stage germ cells from P15 testis. In both wild-type and *Meioc* -/- germ cells, SYCP3 localizes along the entire length of chromosomes, and SYCP1 localizes to regions of synapsis. (E) Immunofluorescence staining for SYCP3 and REC8 in chromosome spreads of wild-type and *Meioc* -/- germ cells from P15 testis. DNA stained by DAPI. In both wild-type and *Meioc* -/- zygotene stage germ cells, SYCP3 and REC8 localize along the entire length of chromosomes. In metaphase-like cells, SYCP3 and REC8 localize, respectively, to the ends of chromosomes and to the condensed chromosomes. This localization of SYCP3 and REC8 is similar to that observed in wild-type metaphase I germ cells adult testes.

Meiotic initiation requires *Stra8*, a retinoic acid-induced, germ cell-specific factor [14,15]. Germ cells in both wild-type and *Meioc*-deficient P10 testes and E14.5 ovaries express STRA8 (Fig 3A). One of the first events following the decision to initiate meiosis is premeiotic DNA replication. To detect DNA replication, we injected the thymidine analog EdU into wild-type and *Meioc*-deficient postnatal male mice, or into pregnant mothers carrying wild-type and *Meioc*-deficient fetal female mice, and harvested gonads two hours later. Both wild-type and *Meioc*-deficient P10 testes and E14.5 ovaries had numerous EdU and STRA8 double-positive cells (Fig 3A), indicating that they are able to undergo premeiotic DNA replication following the decision to enter meiosis.

Next, we examined *Meioc*-deficient germ cells for markers of the chromosomal program of meiotic prophase I, including homologous chromosome synapsis, recombination, and loading of meiotic cohesins.

We first stained for components of the synaptonemal complex: the axial element protein SYCP3, and transverse filament protein SYCP1 (Figs 3B–3D and S8A) [19,20]. In wild-type P15 testis sections and spreads, we observed SYCP3 and SYCP1 staining indicative of the leptotene, zygotene, and pachytene stages of meiotic prophase: SYCP3 staining was thin and thread-like in the leptotene stage, and progressively thickened as chromosomes synapsed through the pachytene stage. SYCP1 localized to synapsed regions of the chromosomes in zygotene and pachytene stage spermatocytes. In *Meioc*-deficient P15 testes, some germ cells exhibited SYCP3 and SYCP1 localization on chromosomes similar to leptotene and zygotene stages, but which were often accompanied by dense aggregates of SYCP3. Many germ cells displayed only SYCP3 aggregates. In the metaphase-like cells, SYCP3 localized to foci at the ends of chromosomes, likely the centromeres. This pattern of SYCP3 localization is similar to that observed in the first meiotic metaphases that normally appear beginning at P20 (Fig 3E) [21]. In wild-type E16.5 ovary sections, most germ cells were in zygotene and pachytene. In *Meioc*-deficient E16.5 ovary sections, no germ cells exhibited zygotene or pachytene-like SYCP3 staining. Instead, *Meioc*-deficient germ cells had either leptotene-like SYCP3 staining with some SYCP3 aggregates, or only SYCP3 aggregates (Figs 3B and S8A).

We next assayed *Meioc*-deficient cells for markers of meiotic recombination. Recombination is initiated by the formation of DNA double-strand breaks (DSBs) that are repaired by meiotic recombinase DMC1 [22,23]. Cells respond to DSBs by phosphorylating the histone variant H2AX, to yield γH2AX [24]. We assessed DSB formation by co-staining for DMC1 and γH2AX alongside SYCP3 in sections and spreads from wild-type and *Meioc*-deficient P15 testes and E16.5 ovaries (Figs 3B, 3C and S8A). In wild-type P15 testes and E16.5 ovaries, we observed DMC1 foci and γH2AX staining indicative of leptotene, zygotene, and pachytene stages of meiosis. In both *Meioc*-deficient P15 testes and E16.5 ovaries, DMC1 foci and γH2AX were also present in leptotene/zygotene-like cells. In metaphase-like cells, DMC1 foci are absent, but γH2AX staining suggests these cells suffer DNA damage.

Finally, we asked if cohesins are loaded onto chromosomes of *Meioc*-deficient germ cells. We immunostained for REC8, a meiotic cohesin [21,25], on spreads of meiotic cells from P15 testes (Fig 3E). In the leptotene/zygotene-like *Meioc*-deficient cells, REC8 localized along the lengths of chromosomes, much as in wild type. In metaphase-like cells, REC8 localizes to the condensed chromosomes, similar to what is observed in meiotic metaphases found in wild-type adult testes (Fig 3E) [21].

Quantification of cell spreads reveals that in P15 *Meioc*-deficient testes, metaphase-like and other abnormal germ cells (such as those with only clumpy SYCP3 staining) comprised about half of all germ cells (S8B Fig). In contrast, no meiotic metaphases were observed in wild-type P15 testes. *Meioc*-deficient testes also contained more leptotene stage germ cells but fewer zygotene stage germ cells than wild-type testes.

In summary, *Meioc*-deficient metaphase-like cells express and correctly localize proteins associated with synapsis and sister chromatid cohesion, demonstrating that the primary defect driving these cells to premature metaphase occurs after they have initiated meiosis. A subpopulation of cells is able to proceed with synapsis, cohesion, and recombination up to the leptotene/zygotene stages. Abby and colleagues focused their attention on the defects in this leptotene/zygotene cell population [18]. However, given our earlier histological analyses showing that metaphase-like cells first arise prior to leptotene and zygotene, it is unlikely that problems in synapsis and recombination cause the premature metaphases. The failure to proceed past the zygotene stage of synapsis and recombination is more likely a secondary consequence of the primary defect driving premature metaphase.

### *Meioc*-deficient testicular and ovarian germ cells form univalent metaphases

Analysis of germ cells spreads showed that in *Meioc*-deficient metaphase-like cells, SYCP3 localized to the centromeres, and REC8 to the condensed chromosomes, similar to wild-type meiotic metaphases (Fig 3C and 3E). However, *Meioc*-deficient metaphase-like cells formed univalents instead of the bivalents formed in wild-type meiotic metaphases. Wild-type metaphase I cells form 20 bivalents, with 40 SYCP3 foci organized into 20 doublets, corresponding to 40 chromosomes organized into 20 homologous pairs. In contrast, *Meioc*-deficient metaphase-like cells retain 40 univalents, with 80 foci organized into 40 doublets, corresponding to 40 paired sister chromatids, with homologous chromosomes unpaired. The doublets of SYPC3 foci in the *Meioc*-deficient metaphase-like cells likely correspond to sister chromatid centromeres that have slightly separated, indicating a failure to maintain cohesion at sister centromeres. We did not observe any bivalents in the *Meioc* mutant amongst testis spreads from three P15 animals.

**Fig 4:**
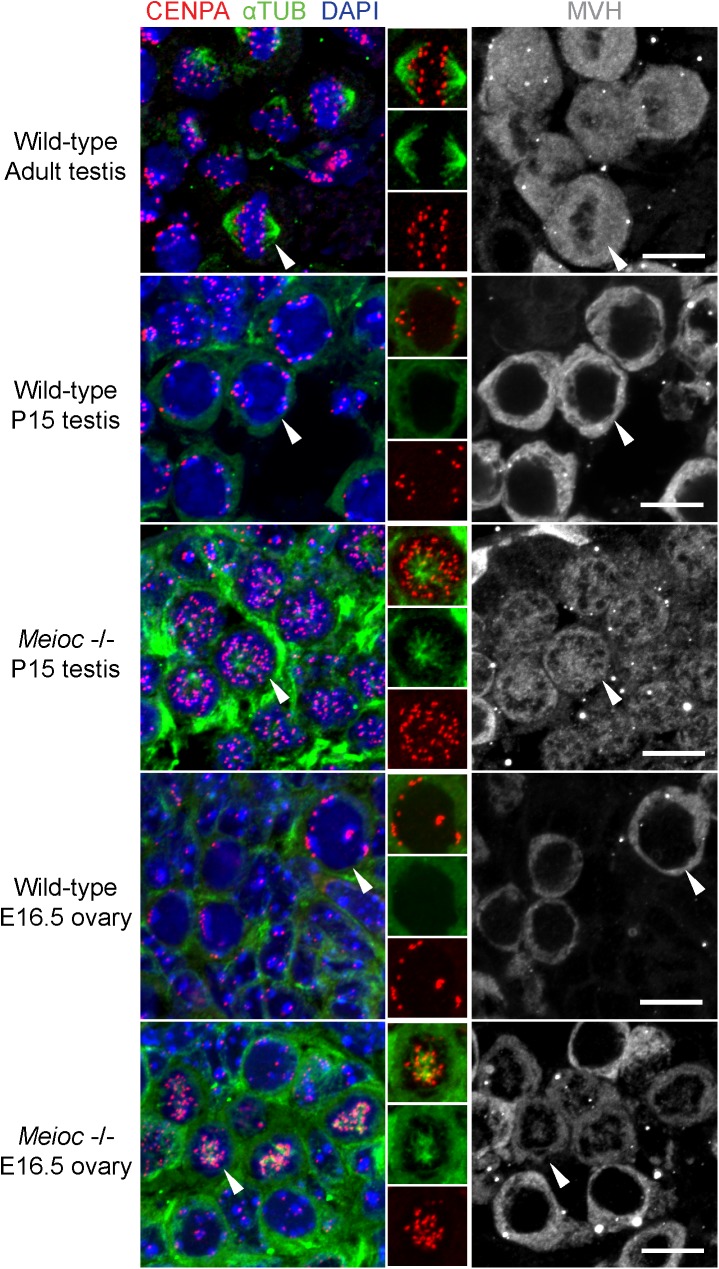
*Meioc*-deficient testicular and ovarian germ cells express molecular markers of metaphase. Immunofluorescence staining for CENPA and α-TUB in wild-type adult testicular germ cells in metaphase I, as well as wild-type and *Meioc* -/- P15 testis and E16.5 ovary sections. Nuclei stained by DAPI; MVH immunostains germ cells. Inset: CENPA and α-TUB staining together, and separately. In wild-type adult testicular germ cells in metaphase I, CENPA localizes to the metaphase plate and a bipolar spindle is formed. In wild-type P15 testis and E16.5 ovary, CENPA localizes to periphery of nuclei in meiotic cells, and no spindle is observed. In *Meioc* -/- P15 testis and E16.5 ovary, CENPA does not localize to periphery of nuclei, and instead localizes to ends of a disorganized, radiating spindle. Scale bar = 10 μm.

We asked if the *Meioc*-deficient cells with univalent chromosomes undergo molecular events associated with metaphase. In germ cells undergoing meiotic metaphase I in adult wild-type testes, chromosomes, visualized via DAPI, align at the equator of the cell to form a metaphase plate. The chromosomes are aligned by a bipolar spindle, formed by α-tubulin-positive microtubules emanating from opposite poles of the cell and attaching to the centromeres, marked by centromeric histone H3 variant CENPA (Fig 4). These features of metaphase are patently absent in wild-type P15 testes and wild-type E16.5 ovaries, where the chromosomes are not yet condensed, and centromeres localize along the nuclear envelope, as previously described [26]. Metaphase-like cells from *Meioc*-deficient P15 testes and E16.5 ovaries assemble a spindle, albeit a disorganized one that appears to emanate from a single pole. Their chromosomes do not assemble on a metaphase plate, and are instead scattered throughout the nucleus. In addition, *Meioc*-deficient metaphase-like germ cells undergo histone H3 phosphorylation and nuclear envelope breakdown, two events associated with wild-type metaphase (S9 Fig). In summary, metaphase-like cells from *Meioc*-deficient mice form spindles, phosphorylate histone H3 and undergo nuclear envelope breakdown much like wild-type meiotic metaphase cells. However, the chromosomes are in univalent rather than bivalent configuration, indicating a failure of the chromosomes to pair, likely as a result of prematurely proceeding to metaphase.

### Aberrant expression of cyclins in meiotic *Meioc*-deficient testicular and ovarian germ cells

To gain insight into the molecular pathways that *Meioc* may regulate so as to extend meiotic prophase I, we performed RNA-seq on whole ovaries from E14.5 wild-type and *Meioc*-deficient fetuses (S1 Table). At this stage, *Meioc*-deficient ovaries did not exhibit TUNEL-positive apoptotic cells, which indicates that programmed cell death had not yet affected the size of the germ cell population (S6 Fig). We observed, in *Meioc*-deficient ovaries, 465 genes expressed at higher levels than wild type (q < 0.01) and 496 genes expressed at lower levels than wild type (q < 0.01); the two sets of genes were enriched for distinct functions (Table 1; S2 Table). Genes expressed at lower levels were enriched for involvement in the meiotic chromosomal program, which we interpreted as reflecting fewer cells entering meiotic prophase I in the mutant. Genes expressed at higher levels were enriched for factors typically associated with the mitotic cell cycle. Previously reported microarray analyses of *Meioc*-deficient gonads identified only 42 differentially expressed genes, of which 38 were expressed at lower levels [18]. Of these 38 genes, half were noted to be associated with meiosis. Those analyses failed to detect genes expressed at higher levels, and thus did not identify the misregulation of mitotic cell cycle factors. Given that RNA-seq provides more sensitivity than microarray analysis [27], our RNA-seq analysis likely reveals a more complete snapshot of transcriptional changes in the absence of *Meioc*.

**Table 1:** Top ten enriched GO categories for genes expressed at lower or higher levels in *Meioc*-/- ovaries.

***Genes expressed at lower levels in Meioc-/- ovaries***		
GO term	Fold Enrichment	Benjamini-corrected p-val
cell cycle process	2.75	9.80E-03
cell cycle	2.22	2.03E-02
meiosis	5.00	3.48E-02
M phase of meiotic cell cycle	5.00	3.48E-02
cell cycle phase	2.68	3.09E-02
meiotic cell cycle	4.88	2.55E-02
cofactor metabolic process	3.29	5.01E-02
meiosis I	7.99	4.59E-02
chromosome organization involved in meiosis	11.75	1.32E-01
synapsis	11.75	1.32E-01
***Genes expressed at higher levels in Meioc-/- ovaries***		
GO term	Fold Enrichment	Benjamini-corrected p-val
cell division	4.90	8.12E-10
mitotic cell cycle	4.76	7.39E-08
cell cycle phase	4.06	7.63E-08
cell cycle	2.96	3.39E-07
cell cycle process	3.50	8.64E-07
nuclear division	4.98	8.71E-07
mitosis	4.98	8.71E-07
M phase of mitotic cell cycle	4.88	1.09E-06
organelle fission	4.80	1.26E-06
M phase	3.95	1.90E-06

We explored the possibility that the premature metaphase entry observed in *Meioc*-deficient germ cells was associated with misregulation of cell cycle factors. Progression through the cell cycle is tightly controlled by cyclical fluctuations in expression of cyclins, which induce oscillatory activation of cyclin-dependent kinases. We therefore examined cyclin expression, focusing on determining whether *Meioc*-deficient germ cells express cyclins typical of mitosis or meiosis.

**Fig 5:**
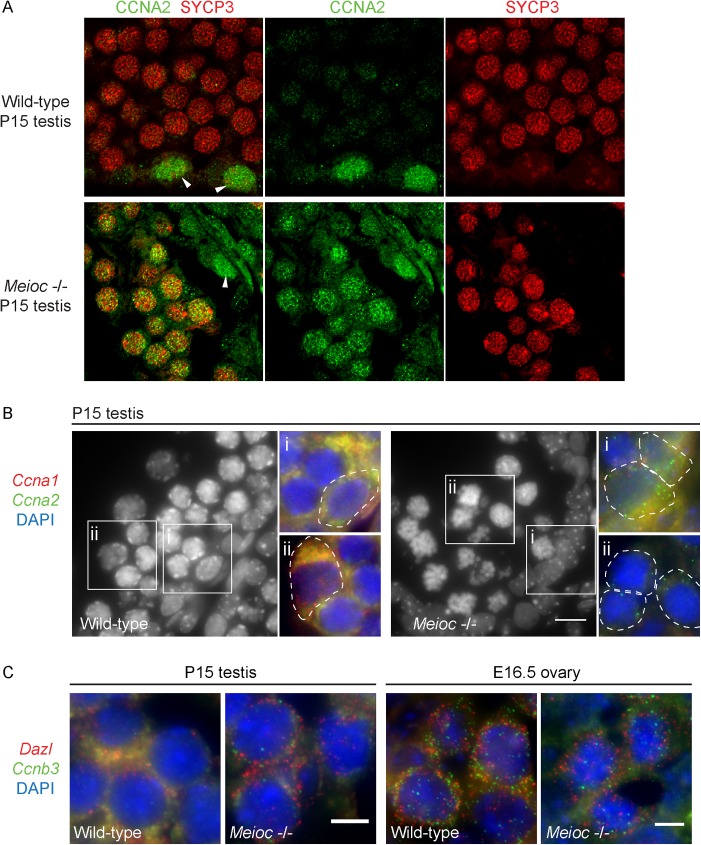
Expression of *cyclin A2* and *cyclin B3* in *Meioc*-deficient adult testis and ovary. (A) Immunofluorescence staining for CCNA2 and SYCP3 in wild-type and *Meioc* -/- P15 testis. In wild-type, CCNA2 is expressed in mitotic spermatogonia (arrowhead), and is not expressed in cells past the leptotene stage of meiosis (SYCP3+ cells at zygotene stage of meiosis). In *Meioc* -/- testes, CCNA2 is misexpressed in SYCP3+ cells at zygotene-like stage. (B) Single molecule FISH staining for *Ccna1* and *Ccna2* in wild-type and *Meioc* -/- P15 testis. Low magnification image: DAPI staining of germ cells in testis tubule. High magnification images: DAPI is in blue, and single cells are outlined. Single transcripts are detected as individual red or green dots; diffuse staining is background. *Ccna2* (green dots) is detected in both wild-type and *Meioc* -/- testes in mitotic spermatogonia, identified by their position at base of tubule and by their nuclear morphology (wild-type and *Meioc* -/-, i). *Ccna2* is additionally detected in *Meioc* -/- germ cells that are in middle of lumen (likely meiotic, or else metaphasic cells, ii). *Ccna1* (red dots) is detected in late pachytene germ cells in wild-type testis, but not in *Meioc* -/- germ cells (wild-type and *Meioc* -/-, ii). (C) Single molecule FISH staining for *Ccnb3* and *Dazl* in late zygotene/early pachytene germ cells from wild-type and *Meioc* -/- P15 testis and E16.5 ovary. *Ccnb3* and *Dazl* are detected in both wild-type and *Meioc* -/- germ cells. Scale bar = 10 μm.

Cyclin A2 (CCNA2), which drives progression through mitotic S and G2-M [28], is expressed in the male germline in mitotic spermatogonia and preleptotene spermatocytes, and is normally down-regulated upon entry into leptotene [29,30]. We immunostained wild-type and *Meioc*-deficient P15 testes for CCNA2, as well as SYCP3, to identify cells in meiotic prophase. We confirmed that in the wild-type, CCNA2 is expressed in mitotic spermatogonia, but not in germ cells that had entered meiotic prophase, as evident by thread-like SYCP3 staining (Fig 5A). In contrast, in *Meioc*-deficient testes, CCNA2 is present in germ cells that exhibit SYCP3 staining typical of leptotene and zygotene. Thus, in *Meioc*-deficient testes, testicular germ cells in meiotic prophase aberrantly express CCNA2.

Cyclin A1 (CCNA1) is thought to replace CCNA2 during the meiotic cell cycle: it is expressed in meiotic spermatocytes from late pachytene through metaphase, and is required to initiate meiotic metaphase [31,32]. Using single molecule fluorescent in situ hybridization (smFISH), we observed *Ccna1* mRNA expression in wild-type P15 testes in late pachytene cells, but not spermatogonia, which instead expressed *Ccna2* (Fig 5B). In *Meioc*-deficient P15 testes, we failed to observe *Ccna1* expression in either meiotic or metaphase-like germ cells.

Cyclin Bs are essential for the G2/M transition [28]. Cyclin B1 and B2 (CCNB1, CCNB2) are expressed in both mitotically and meiotically dividing cells. In contrast, cyclin B3 (CCNB3) is expressed only during leptotene and zygotene in both males and females, and forms kinase-deficient complexes with CDK2, raising the possibility that CCNB3 could be inhibiting precocious cell cycle progression during early meiotic prophase I [33,34]. Using smFISH, we observed *Ccnb3* expression in meiotic cells of wild-type P15 testes and E16.5 ovaries as expected (Fig 5C), and also in meiotic germ cells from *Meioc*-deficient testes and ovaries.

In summary, we found that *Meioc*-deficient meiotic germ cells do not exclusively express either mitosis or meiosis-specific cyclins. They express meiosis-specific CCNB3, suggesting that they have initiated the meiotic cell cycle program, but they also aberrantly express CCNA2, which should be down-regulated during meiosis. Misexpression of CCNA2, accompanied by the broad up-regulation of genes associated with the mitotic cell cycle, leads us to conclude that although *Meioc*-deficient germ cells can initiate the meiotic chromosomal program, they fail to properly transition from a mitotic to meiotic cell cycle program. Based on these novel findings, not reported by Abby et al. [18], we propose that mis-regulation of the cell cycle is the primary cause of premature metaphases in the absence of *Meioc*.

### MEIOC interacts with the mouse homolog of BGCN, a translational regulator in fly

**Fig 6:**
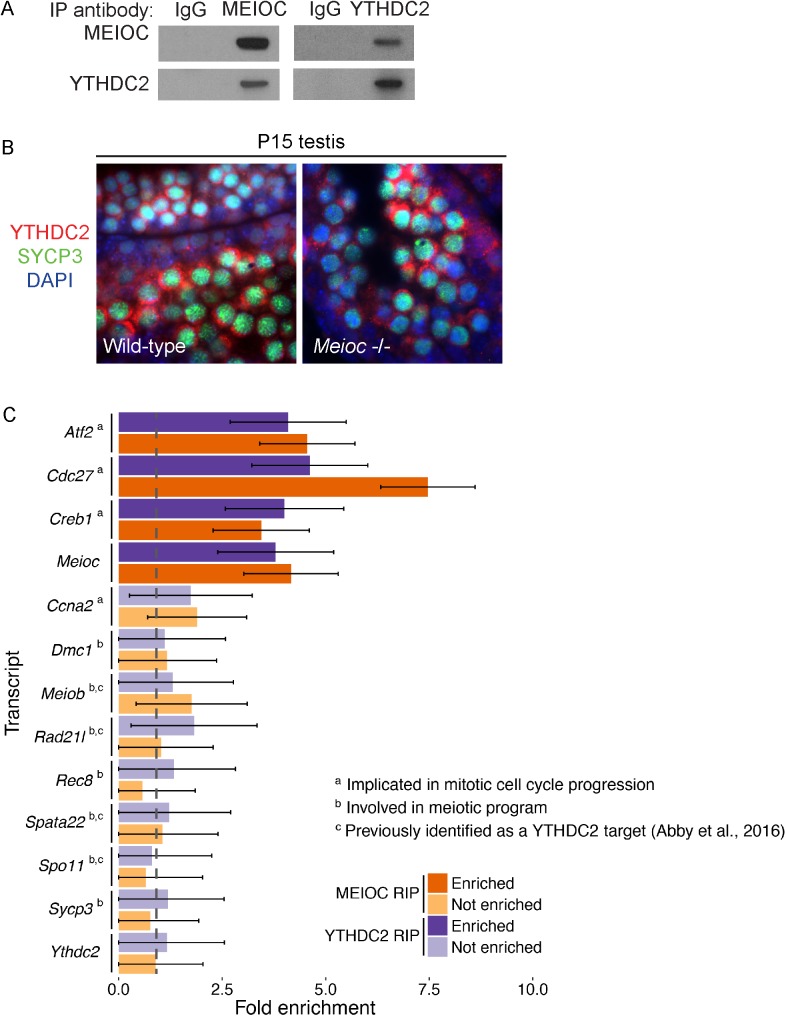
MEIOC co-immunoprecipitates with YTHDC2 and cell cycle-associated transcripts but not meiosis-specific transcripts. (A) Immunoprecipitation (IP) performed with anti-MEIOC or anti-YTHDC2 antibody and IgG control from adult testis lysates. IP was followed by immunoblotting with either the anti-MEIOC antibody, or anti-YTHDC2 antibody. MEIOC and YTHDC2 were detected specifically in immunoprecipitation with either anti-MEIOC or anti-YTHDC2 antibody. (B) Immunofluorescence staining for YTHDC2 and SYCP3 in wild-type and *Meioc* -/- P15 testis. Nuclei stained by DAPI. YTHDC2 is expressed at comparable levels in zygotene and zygotene-like cells from both wild-type and *Meioc* -/- testes, respectively. (C) Fold enrichment for MEIOC-specific binding in P15 testis and YTHDC2-specific binding in P20 testis. For MEIOC, targets were identified via MEIOC RIP-seq and total RNA-seq analyses of wild-type and *Meioc* -/- testes as well as IgG RIP-seq from wild-type testes. For YTHDC2, targets were identified via YTHDC2 RIP-seq, IgG RIP seq, and total RNA-seq analyses of wild-type testes. Statistically significant enrichment was identified based on FDR < 0.05, FPKM > 1, and fold change > 3 for MEIOC, or fold change > 2 for YTHDC2. Of the transcripts that were enriched, some have been implicated in mitotic cell cycle progression. Transcripts that were not enriched were selected for analysis based on functions in the cell cycle (*Ccna2*), the canonical meiotic chromosomal program (*Dmc1*, *Rec8*, *Sycp3*), or previous reports of interaction with YTHDC2 (*Meiob*, *Rad21l*, *Spata22*, *Spo11*). Error bars represent standard error. Dashed grey line marks fold change of 1.

To gain insight into how MEIOC functions at the molecular level to prevent premature exit from meiotic prophase I, we determined MEIOC’s binding partners by performing an immunoprecipitation for MEIOC from testis lysates. Using quantitative mass spectrometry analysis, we identified one protein as interacting with MEIOC: YTHDC2 (enrichment over MEIOC immunoprecipitation in *Meioc*-deficient testes > 1.5, unique peptides >1; Table 2, S3 Table). We confirmed the interaction between MEIOC and YTHDC2 by immunoprecipitating each protein from adult testes and immunoblotting for the other (Fig 6A). MEIOC interaction with YTHDC2 was also previously observed [18].

**Table 2:** Identification of MEIOC-interacting proteins by quantitative mass spectrometry.

Protein	Average TMT values relative to one replicate of MEIOC IP from *Meioc* -/- lysate (SD)			Number of unique peptides selected for fragmentation*
	IgG IP from wild-type lysate	MEIOC IP from wild-type lysate	MEIOC IP from *Meioc* -/- lysate	
MEIOC / GM1564	0.866 (0.033)	4.055 (0.673)	1.136 (0.193)	4
YTHDC2	0.631 (0.100)	2.028 (0.449)	1.069 (0.098)	9

*Sequences of unique peptides listed in S3 Table.

TMT quantification was obtained for two biological replicates in each of three immunoprecipitation conditions: (A) wild-type (C57BL/6) lysate with IgG antibody, (B) wild-type lysate with MEIOC antibody, or (C) *Meioc* -/- lysate with MEIOC antibody. Values were normalized to the signal from one replicate in condition C and then averaged across each condition. Shown here are all proteins represented by two or more peptides with a relative TMT value greater than 2 in condition B relative to condition C.

YTHDC2 contains multiple domains that interact with nucleic acid—specifically, an R3H domain, an RNA helicase domain, and a YTH domain [35–37]—but its molecular function in mammalian cells remains poorly characterized. To gain insight into the function of YTHDC2, we looked for YTHDC2 orthologs in other species. We identified YTHDC2 orthologs in almost all metazoans examined (S10 Fig). In *Drosophila melanogaster*, the ortholog of mouse YTHDC2 is BGCN, which physically interacts with a partner, BAM, to regulate translation in germ cells [38]. Considering that we find no ortholog of MEIOC in the *Drosophila* genome (S1 Fig), mouse MEIOC may be interacting with YTHDC2 to perform a role analogous to that of BAM with BGCN in *Drosophila*. Based on this hypothesis, we might expect similar phenotypes in *Meioc-*deficient and *Ythdc2-*deficient mice. *Ythdc2-*deficient male mice exhibit striking similarities to the *Meioc-*deficient mice: in both mutants, germ cells initiate but do not complete meiosis; instead, numerous abnormal metaphase-like cells are observed (A. Bailey, D. de Rooij, and M. Fuller, personal communication).

To determine if YTHDC2 protein expression is regulated by MEIOC, we immunostained wild-type and *Meioc*-deficient P15 testes for YTHDC2 (Fig 6B). In both wild-type and *Meioc*-deficient testes, YTHDC2 was present in the cytoplasm of meiotic germ cells, including leptotene, zygotene, and pachytene cells in the wild-type, and leptotene/zygotene-like cells in the mutant. Thus, in contrast to previous reports [18], we found that YTHDC2 expression is not dependent on *Meioc*.

### MEIOC interacts with cell cycle-associated transcripts but not meiosis-specific transcripts

Given that YTHDC2 and MEIOC proteins localize to the cytoplasm, and that YTHDC2 contains multiple domains that interact with nucleic acid (specifically, an R3H domain, an RNA helicase domain, and a YTH domain) [35–37], we hypothesized that a YTHDC2/MEIOC complex binds to and post-transcriptionally regulates mRNA, like the *Drosophila* BGCN/BAM complex. Based on the observations that *Meioc*-deficient germ cells exhibit precocious progression into a metaphase-like state and misexpress cell cycle transcripts and mitotic cyclin CCNA2, we further hypothesized that this YTHDC2/MEIOC complex regulates transcripts involved in mitotic cell cycle progression.

We therefore investigated the transcripts to which both MEIOC and YTHDC2 bind via RNA immunoprecipitation and sequencing (RIP-seq). We performed MEIOC RIP-seq in wild-type P15 testes, along with the following controls: MEIOC RIP-seq in *Meioc*-deficient P15 testes, IgG RIP-seq controls in wild-type P15 testes, and RNA-seq from both wild-type and *Meioc*-deficient testes to control for changes in mRNA abundances in wild-type and *Meioc*-deficient testes. We performed YTHDC2 RIP-seq in P20 testes using two independent YTHDC2 antibodies, along with the following controls: IgG RIP-seq in wild-type P20 testes, and RNA-seq in wild-type testes. We identified 626 transcripts that were enriched in immunoprecipitation with MEIOC (fold change > 3, FDR < 0.05, expressed at FPKM > 1, S4 Table), and 80 transcripts enriched in immunoprecipitation with YTHDC2 (fold change > 2, FDR < 0.05, expressed at FPKM > 1, S4 Table). Of these, 67 transcripts were identified as both MEIOC and YTHDC2 targets (a subset of results shown in Fig 6C). We validated a sampling of the MEIOC and YTHDC2 targets by RIP followed by quantitative PCR (qPCR; S11 Fig). While *Ccna2* was not a direct target of MEIOC or YTHDC2, bound transcripts included other cell-cycle related transcripts such as *Cdc27*, a component of the anaphase promoting complex [39], as well as *Creb1* and *Atf2*, transcription factors that can upregulate the expression of *Ccna2* [40–42]. In addition, both MEIOC and YTHDC2 interact with the *Meioc* transcript itself, but not with *Ythdc2* transcript.

In contrast to our model of the MEIOC/YTHDC2 complex as a regulator of meiotic prophase I exit, Abby and colleagues suggested that MEIOC and YTHDC2 function to stabilize transcripts involved in the chromosomal program of meiosis [18]. This conclusion was based, in part, on RIP data indicating that YTHDC2 bound four transcripts essential to the chromosomal program (*Spata22*, *Spo11*, *Meiob*, and *Rad21L*) [18]. This hypothesis predicts that MEIOC should also interact with these transcripts. We found no evidence, by either RIP-seq or RIP-qPCR, that MEIOC or YTHDC2 interacts with these transcripts (Fig 6C, S11 Fig). Furthermore, we could not demonstrate enrichment for additional canonical transcripts in the meiotic chromosomal program, such as *Dmc1*, *Rec8*, and *Sycp3* (Fig 6C; S4 Table), which were not identified as YTHDC2 targets by Abby and colleagues [18].

To determine whether the MEIOC/YTHDC2 complex promotes or inhibits expression of its targets, we returned to our RNA-seq dataset from E14.5 wild-type and *Meioc*-deficient ovaries. Given the remarkable similarity of *Meioc*-deficient phenotypes in males and females, we hypothesized that MEIOC/YTHDC2’s targets from the testis would also be differentially expressed in the fetal ovary. We therefore compared MEIOC/YTHDC2’s shared targets to our RNA-seq dataset from E14.5 wild-type and *Meioc*-deficient ovaries (S1 Table). Of the 67 MEIOC- and YTHDC2-bound mRNAs identified in the testis, 65 were expressed (FPKM > 1) in the fetal ovary. Of these 65 MEIOC- and YTHDC2-bound transcripts, 28 (43%) were expressed differentially between E14.5 wild-type and *Meioc*-deficient ovaries. With the exception of the *Meioc* transcript itself, all 27 of these differentially expressed mRNAs were present at higher levels in the absence of MEIOC (S5 Table), suggesting that MEIOC and YTHDC2 destabilize their target mRNAs. These differentially expressed targets included the mitotic cell cycle regulators *Atf2*, *Cdc27*, and *Creb1*. Not all MEIOC/YTHDC2-bound mRNAs were observed to be differentially expressed. This may be because most MEIOC/YTHDC2-bound mRNAs were expressed in gonadal somatic cells as well as in germ cells, which may obscure differential expression signals in RNA-seq data from whole gonads. Additionally, our MEIOC and YTHDC2 RIP experiments were performed using testis tissue, while our RNA-seq data was derived from fetal ovary; though they overlap, the sets of genes targeted by MEIOC and YTHDC2 in testis and ovary may not be identical.

In summary, we found that MEIOC and YTHDC2 bind transcripts that regulate the mitotic cell cycle, likely resulting in their destabilization. These observations are consistent with our hypothesis that MEIOC facilitates the switch from a mitotic to a meiotic cell cycle program. We find no evidence that MEIOC interacts with transcripts of the meiotic chromosomal program, and thus no reason to believe that it directly stabilizes such transcripts, as recently proposed by Abby and colleagues [18].

## Discussion

An extended prophase I is a conserved feature of meiosis, and is critical for enabling completion of meiotic chromosomal events in yeast [2]. Our analyses of *Meioc*-deficient mice identify *Meioc* as a critical factor required for this extended prophase I in mice: in the absence of *Meioc*, both testicular and ovarian germ cells can initiate meiosis and embark on meiotic prophase I, but fail to progress past the zygotene stage. Instead, *Meioc*-deficient cells proceed precociously to metaphase. Our studies demonstrate that *Meioc* is required for an extended meiotic prophase I in mice, and reveal the extended prophase I as a critical and actively regulated feature of meiosis in a vertebrate system.

**Fig 7:**
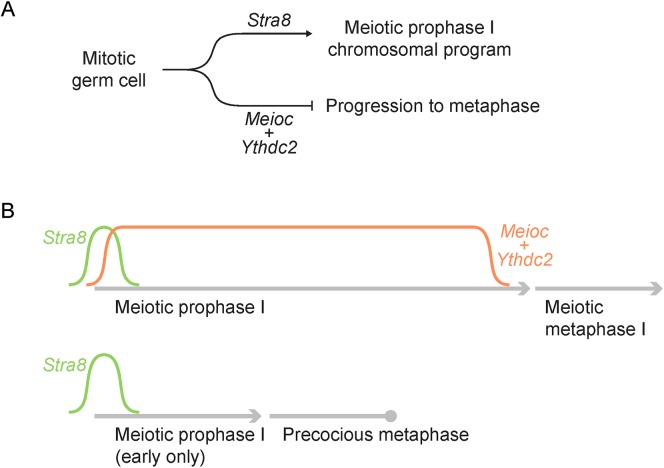
A proposed model of *Meioc*’s role in meiosis. (A) Mitotic germ cells transition into meiosis via expression of *Stra8*, which upregulates the meiotic chromosomal program. At the same time, *Meioc* and *Ythdc2* are required during meiotic prophase to inhibit progression into metaphase, thereby allowing meiotic prophase to proceed normally. (B) *Stra8* is expressed in early meiotic prophase, while *Meioc* is expressed throughout meiotic prophase. In wild-type germ cells, *Meioc* is downregulated before progression into metaphase. In the absence of *Meioc*, meiotic prophase is abbreviated, resulting in a precocious attempt at metaphase.

We posit that meiotic prophase I is comprised of various meiosis-specific subprograms, including a chromosomal program wherein chromosomes synapse and recombine, and a coordinately regulated cell cycle program that extends prophase I for the duration of the chromosomal program. We propose that when the chromosomal program of meiosis is initiated, the corresponding cell cycle program must be simultaneously implemented (Fig 7). Our previous findings demonstrated that *Stra8* is required for the meiotic chromosomal program [14,15]. Our present findings lead us to propose that *Meioc* is simultaneously required to promote the meiotic cell cycle program.

How can a germ cell ensure that it exits prophase into metaphase only when the meiotic chromosomal program is complete? We reasoned that the germ cell must transition from a mitotic cell cycle program (that of necessity is independent of the meiotic chromosomal program) to a meiotic cell cycle program in which prophase exit is dependent on meiotic chromosomal checkpoints. We hypothesize that *Meioc* is required for this transition. This model predicts that in the absence of *Meioc*, a germ cell that has already expressed key meiotic regulators (such as STRA8) and meiotic chromosomal proteins (such as SYCP3) will continue to run a mitotic cell cycle program. Due to an active mitotic cell cycle program, the meiotic cell will proceed to metaphase on a mitotic schedule, independent of the meiotic chromosomal checkpoints. It was previously observed that leptotene/zygotene stage spermatocytes are not competent to enter metaphase upon stimulation with okadaic acid [43]; this is likely because in wild-type cells, exit from prophase into metaphase is strictly dependent on the meiotic chromosomal checkpoints. In contrast, in *Meioc*-deficient germ cells, a persistent mitotic cell cycle program renders the cell cycle independent of the meiotic chromosomal events, and drives cells into metaphase as early as preleptotene, or shortly thereafter. Consistent with this idea of cell cycle mis-regulation, *Meioc*-deficient spermatocytes that have initiated meiotic prophase I misexpress Cyclin A2, which is normally expressed in mitotic spermatogonia and down-regulated by leptotene of meiotic prophase I.

A second possibility is that *Meioc* functions to establish a checkpoint for exit from meiotic prophase I. However, if lack of a checkpoint led to premature resumption of the meiotic cell cycle, we might expect that the cell cycle resumed would be meiotic in nature, and thus primarily driven by cyclins typically expressed during the meiotic cell cycle, such as Cyclin A1. Instead, we find that Cyclin A2, not Cyclin A1, is expressed in *Meioc*-deficient germ cells. Therefore, MEIOC appears to govern the transition from a mitotic to a meiotic cell cycle program, in part or in whole by suppressing the mitotic program.

An alternate model has been proposed by Abby et al., wherein *Meioc* is required for stabilization of meiotic transcripts, such as those required for the chromosomal program [18]. In their model, the failure to stabilize these transcripts leads to lack of sufficient proteins required for the chromosomal events of meiosis, thus forcing cells to switch prematurely to metaphase. We find this model unsatisfying for the following reasons. First, we find no evidence that MEIOC and YTHDC2 bind transcripts that function in the chromosomal program. The conditions used for RIP experiments may explain the difference between our results and those of Abby et al. For immunoprecipitation of RNA, we used lysis conditions without reducing agents in order to maintain proteins’ disulfide bonds. By contrast, Abby et al. used mild reducing conditions that could have relaxed disulfide bonds and potentially altered the proteins and transcripts with which YTHDC2 interacted. We propose that the non-reducing conditions used in this study are more likely to have captured the in vivo interactions of MEIOC and YTHDC2. In addition, the model proposed by Abby et al. does not explain how a failure to stabilize transcripts of the meiotic chromosomal program results in premature metaphase. In the vast majority of knock-outs of genes required for the meiotic chromosomal program (e.g. *Dmc1*), germ cells arrest in meiosis and proceed to apoptosis, rather than attempting precocious metaphase [22,23].

Understanding the molecular function of MEIOC would aid in distinguishing these two alternative models. Our genetic and biochemical analyses suggest a role for MEIOC in post-transcriptional regulation of transcripts implicated in the cell cycle. First, the phenotype of *Meioc*-deficient mice is highly similar to that of male mice deficient for the mouse ortholog of *Bgcn* (A. Bailey, D. de Rooij, and M. Fuller, personal communication). *Drosophila* BGCN is an RNA helicase that acts in concert with an interacting partner, BAM, to repress translation in *Drosophila* germ cells [38,44,45]. The shared phenotype between mouse *Meioc* and *Ythdc2* suggests that they may act as interacting partners to regulate translation in the mouse germline, similar to BAM/BGCN in the fly. A putative mouse ortholog of fly *bam* had been previously identified, but mice lacking this gene exhibited no viability or fertility defects [46]. *Meioc*, while not orthologous to *Drosophila bam*, may be its functional analog in the mouse. Notably, we failed to identify an ortholog of *Meioc* in *Drosophila*, further supporting the notion that mouse MEIOC and *Drosophila* BAM substitute for each other in the two species. Consistent with the hypothesis that MEIOC and YTHDC2 function together, we find evidence that MEIOC physically interacts with YTHDC2. Further, MEIOC and YTHDC2 interact with overlapping sets of transcripts. These transcripts include genes associated with the mitotic cell cycle, but not with meiosis, bolstering our model that a MEIOC/YTHDC2 complex post-transcriptionally regulates transcripts associated with cell cycle progression. Transcripts that interact with MEIOC and YTHDC2 are up-regulated in the absence of MEIOC, suggesting that MEIOC/YTHDC2 functions to destabilize their target mRNAs. While MEIOC’s PF15189 domain remains uncharacterized, YTHDC2 contains multiple domains that interact with nucleic acid. These domains include the R3H domain that binds single-stranded nucleic acid [36]; the DEAH box helicase domain that unwinds nucleic acids [35]; and the YTH domain that recognizes post-transcriptionally modified N6-methyladenosine (m6A) on RNA [37]. In particular, RNA helicases can regulate the stability of target transcripts by interacting with proteins that directly influence RNA stability/degradation, such as decapping enzymes, deadenylation complexes, and ribonucleases [35]. Helicases can further affect RNA stability by unfolding the RNA to make it accessible to these enzymes [35]. However, we do not yet know the extent to which these domains are active in the YTHDC2 protein, and how MEIOC may contribute to their activity. The precise molecular mechanism of MEIOC/YTHDC2 activity, and consequences for target transcripts, remain to be determined.

Mouse MEIOC and YTHDC2, and their *Drosophila* counterparts *bam* and *bgcn*, appear to have similar roles in gametogenesis based on post-transcriptional regulation of transcripts. However, the details of regulation differ between species, and even between sexes. Whereas MEIOC and YTHDC2 appear to regulate the meiotic cell cycle program, *bam* and *bgcn* function at earlier stages of *Drosophila* gametogenesis, prior to the decision to initiate meiosis. In *Drosophila* males, *bam* and *bgcn* are required for spermatogonia to cease proliferation and initiate spermatocyte differentiation and meiosis [47,48]. In the female, *bam* and *bgcn* function earlier to initiate the transit amplifying divisions [49,50]. Correspondingly, their target transcripts differ between the *Drosophila* sexes: BAM and BGCN repress *mei-P26* translation in the male, but not in the female [38]. Conversely, BAM represses translation of *nanos* in the female but not the male [44]. Furthermore, *mei-P26* and *nanos* are not components of the cell cycle program. Thus, while the involvement of the bam-bgcn and MEIOC-YTHDC2 complexes in gametogenesis via post-transcriptional regulation is conserved, their time of action, as well as the targets of translational repression, may vary according to sex and species.

A common pathway induces both initiation of the chromosomal program of meiotic prophase I, as well as *Meioc* expression, thus genetically linking the meiotic chromosomal program with the meiotic cell cycle program. We previously demonstrated through an in vivo genetic knock-out mouse model that *Stra8* is required for initiation of the meiotic chromosomal program in both ovarian and testicular germ cells [14,15]. More recently, further in vivo studies of *Stra8*-deficient ovaries showed that *Stra8* is required for full induction of *Meioc* expression: *Meioc* expression is 4-fold higher in wild-type fetal ovaries than in *Stra8*-deficient ovaries [17]. Since *Stra8* is induced by RA [10,11], *Meioc* expression in fetal ovarian germ cells is thus also at least partially dependent on RA signaling. Contrary to these results, Abby et al. concluded that *Meioc* expression is completely independent of RA signaling in both ovarian and testicular germ cells, based on data from fetal gonads cultured with RA or an RAR inverse agonist as well as postnatal testes from pups exposed to the RAR inverse agonist [18]. This discrepancy in results in ovarian germ cells suggests that the in vivo genetic model may more accurately reflect the endogenous biology than a culture system, especially when dealing with a relatively modest (4-fold) change in gene expression. Therefore, similar in vivo examination of whether RA and *Stra8* contribute to *Meioc* expression in testicular germ cells is still needed.

Our study leads us to propose that successful meiosis in mice requires coordination of a meiosis-specific cell cycle program with the elaborate chromosomal program of prophase I. Further studies will elucidate how *Meioc*, in partnership with *Ythdc2*, promotes the transition to a meiosis-specific cell cycle program at the time germ cells initiate the meiotic chromosomal program.

## Materials and methods

### Ethics statement

All experiments involving mice were performed in accordance with the guidelines of the Massachusetts Institute of Technology (MIT) Division of Comparative Medicine, which is overseen by MIT’s Institutional Animal Care and Use Committee (IACUC). The animal care program at MIT/Whitehead Institute is accredited by the Association for Assessment and Accreditation of Laboratory Animal Care, International (AAALAC), and meets or exceeds the standards of AAALAC as detailed in the Guide for the Care and Use of Laboratory Animals. The MIT IACUC approved this research (no. 0714-074-17).

### Generation of anti-MEIOC antibody

A polyclonal antibody against MEIOC was raised in rabbits against C-terminal peptide CHESINSSNPMNQRGETSKH (YenZym Antibodies, LLC), and affinity purified using the antigenic peptide (SulfoLink Immobilization Kit for Peptides, ThermoScientific).

### Generation of *Meioc* mutant alleles

The *Meioc* gene was targeted for homologous recombination in v6.5 embryonic stem (ES) cells with a targeting vector for a knockout-first allele of *Meioc* (obtained from the Knockout Mouse Project Repository, vector PG00048_X_6_E03) (S4 Fig). Resultant colonies were tested for correct integration by Southern blot analysis of a KpnI/XhoI restriction digest. Three independent, verified ES cell clones were injected into C57BL/6 recipient blastocysts, and germline transmission was obtained with all three clones. The ‘knockout-first’ allele is denoted 3lox or 3L as it retains 3 loxP sites. In the 3lox allele, the open reading frame is disrupted by the active lacZ reporter. The 3lox allele was subject to Flp recombination by breeding mice bearing the 3lox allele to ACTB:FLPe B6J mice (Jackson laboratory no. 005703). The resultant allele is a conditional allele, denoted 2lox or 2L. The *lacZ* and *Neo* genes are excised, leaving exon 3 flanked by loxP sites. The 2lox allele was subject to Cre recombination by breeding mice bearing the 2lox allele to *Mvh*^*Cre-mOrange*^ mice [51]. The resultant allele is a knockout allele, denoted 1lox, 1L or *Meioc* -. Cre recombination excises exon 3, and is predicted to result in a frame shift and generate a premature stop codon subsequent to exon 2. All three alleles were genotyped by PCR (detailed in S4 Fig).

### Mice and sample collection

We analyzed both *Meioc* 3L/3L and *Meioc*-deficient (*Meioc* -/-; *Meioc* 1L/1L) mice. *Meioc* 3L/3L and *Meioc*-deficient mice or embryos were generated by heterozygote matings. For wild-type controls, we used littermates that were either heterozygote for the mutant and wild-type allele or homozygous for the wild-type allele. *Meioc* 3L/3L mice were of mixed 129S4 and C57BL/6 background. *Meioc*-deficient mice were backcrossed to the C57BL/6 strain for at least 5 generations; all data shown in figures are from mice 5 to 7 generations backcrossed.

### EdU incorporation

Mice, or pregnant mothers, were injected with 4μg/μl of EdU dissolved in PBS, for a final dose of 20μg/g. Samples were collected 2 h after EdU injection.

### Histology

Testes were fixed overnight in Bouin’s solution, embedded in paraffin, sectioned, and stained with hematoxylin and eosin. Sections were examined using a light microscope, and germ cell types were identified by their location, nuclear size, and chromatin pattern (Russell et al., 1990).

### Immunostaining of sections

Postnatal or adult testes, or embryonic ovaries, were fixed one of three ways: in 4% paraformaldehyde (PFA) overnight followed by embedding in paraffin, in Bouins solution for 2 h followed by embedding in paraffin, or in 4% PFA for 1 h following by freezing in OCT (Sakura Finetek, Torrance, CA). Paraffin or frozen blocks were sectioned. Paraffin sections were dewaxed, rehydrated, and subject to antigen retrieval by heating in citrate buffer (10mM sodium citrate, 0.05% Tween 20, pH6.0). Frozen sections were thawed and washed in PBS. Sections were then blocked in 5% normal donkey serum, incubated with primary antibodies at 4°C overnight, washed with PBS, incubated with the secondary antibody at room temperature for 1 h, and washed with PBS. Details for primary antibodies and their corresponding fixation and incubation conditions are detailed in S6 Table. For fluorescent detection, fluorophore-conjugated secondary antibodies were used at 1:250 (Jackson Immunoresearch Laboratories or Invitrogen), and sections were mounted in ProLong Gold Antifade reagent with DAPI (Thermo Fisher Scientific). For colorimetric detection, ImmPRESS peroxidase-conjugated secondary antibodies were used (Vector Laboratories), followed by detection using DAB substrate (Vector Laboratories). TUNEL staining was performed on PFA-fixed sections embedded in paraffin using the DeadEnd Colorimetric TUNEL System (Promega) according to the manufacturer’s intstructions. Slides were then counterstained with hematoxylin, dehydrated, and mounted in Permount (Thermo Fisher Scientific). EdU was detected as per manufacturer’s protocol (Click-iT EdU Alexa Fluor 488 Imaging Kit) after secondary antibody incubation and wash.

### Immunostaining of chromosome spreads

Spreads were prepared from male and female meiotic germ cells as previously described [52] with some modifications. Male germ cells in suspension were obtained by mechanically disrupting seminiferous tubules. Germ cells were spun down and resuspended in hypobuffer (30mM TrisHCl pH8.2, 50mM sucrose, 17mM sodium citrate) for 7 min at room temperature, then spun down again and resuspended in 100mM sucrose. Cell suspensions were placed on slides wetted with 1% PFA/0.15% TritonX-100. Female germ cells were obtained by first incubating embryonic ovaries in hypobuffer for 15 min, then mechanically disrupting the ovaries in 100mM sucrose. Dispersed cells were then placed on slides wetted with 1% PFA/0.2% TritonX-100. In both cases, slides were air dried, washed in 0.4% Photo-Flo, and stored at -80C until use. For immunofluorescence staining, frozen sections were thawed and washed in PBS. Sections were then blocked in 3% BSA/1% normal donkey serum/0.05% Triton-X, incubated with primary antibodies at 4°C overnight, washed with PBS, incubated with the secondary antibody at room temperature for 1 h, and washed with PBS. Detailed information on primary antibodies and incubation conditions is provided in S6 Table. Fluorophore-conjugated secondary antibodies were used at 1:250 (Jackson Immunoresearch Laboratories or Invitrogen), and sections were mounted in ProLong Gold Antifade reagent with DAPI (Life Technologies).

### Single molecule fluorescent in situ hybridization

Probe design, synthesis, and coupling were as previously described [53]. Probe sequences are provided in S7 Table. Samples were prepared and hybridization performed as previously described [17,53]. Germ cells were identified by smFISH for *Dazl* and/or nuclear morphology by DAPI staining.

### RNA-seq

We performed RNA-seq on whole ovaries dissected away from mesonephros from E14.5 wild-type and *Meioc* 3L/3L fetuses. Each genotype was represented by three biological replicates of one pair of ovaries each. Total RNA (~1 μg) was extracted from ovaries using Trizol (Invitrogen) according to the manufacturer’s protocol. Libraries were prepared using the Illumina TruSeq RNA Sample Preparation Kit. Libraries were multiplexed and sequenced on the Illumina HiSeq 2000 platform to obtain 40-base-pair single reads. RNA-seq data have been deposited in NCBI GEO under accession number GSE90702 and NCBI SRA under accession number SRP094112. Reads were aligned to the mouse genome (mm10) using TopHat v2.0.11 using default settings, and differential expression analysis was performed using Cufflinks v.2.2.1 [54] with the RefSeq transcript annotation. Enriched GO categories were identified using DAVID [55].

### Immunoprecipitation for immunoblotting and mass spectrometry

To prepare lysates for immunoprecipitation followed by immunoblotting, one testis from a 3-month-old C57BL/6 male was homogenized in lysis buffer (25mM Tris-HCl pH7.5, 150mM NaCl, 1.5mM MgCl_2_, 1mM dithiothreitol (DTT), 0.4% Triton X-100) supplemented with EDTA-free protease inhibitor (Roche Diagnostics) and 250U Benzonase nuclease (EMD Millipore), incubated at 4°C with rotation for 30 min, and then centrifuged at 20,000 *g* for 15 min at 4°C. For immunoprecipitation, the soluble lysate from each testis was pre-cleared for 2 h at 4°C with Dynabeads Protein G (Thermo Fisher Scientific) prior to a 4°C overnight incubation with antibody-bound Dynabeads. Beads were prepared by three brief washes in PBS with 0.1% Tween 20 (PBST) followed by resuspension in PBST and incubation with 5 μg of anti-MEIOC antibody (antibody generation described above) or normal rabbit IgG (Santa Cruz Biotechnology) for 2 h at room temperature. Following the overnight incubation, beads were washed three times with lysis buffer containing 150mM NaCl and transferred to a new tube.

To prepare lysates for mass spectrometry, immunoprecipitations were performed as described above with slight modifications: lysates were prepared from testes of P15 mice, and antibodies were crosslinked to the beads by a 30 min incubation with 5mM bis(sulfosuccinimidyl)suberate. Immunoprecipitations were performed in one of three conditions: wild-type (C57BL/6) lysate with IgG antibody, wild-type lysate with MEIOC antibody, or *Meioc*-deficient lysate with MEIOC antibody. Each condition was represented by two biological replicates, with one testis pair per replicate. The immunoprecipitates were washed three times in wash buffer (25mM Tris-HCl pH7.5, 150mM NaCl, 1.5mM MgCl_2_, 1mM DTT), then washed twice with PBS.

### Immunoblotting

Immunoprecipitated proteins were denatured in sample buffer for 10 min at 70°C, resolved on a NuPAGE 4–12% Bis-Tris gel (Thermo Fisher Scientific), and transferred to a nitrocellulose membrane. The membrane was blocked in 5% BSA/Tris-buffered saline containing 0.1% Tween-20 (TBST) for 1 h at room temperature, incubated overnight at 4°C with a primary antibody solution prepared in 5% BSA/TBST, and incubated for 1 h at room temperature with a 1:5,000 dilution of peroxidase-conjugated anti-rabbit IgG (Jackson Immunoresearch) prepared in 5% BSA/TBST. Proteins on the membrane were detected by the addition of Lumi-Light Western Blotting Substrate (Roche). Antibodies used for immunoblotting were MEIOC (1:2,000) and YTHDC2 (1:1,000; Bethyl Laboratories A303-026A).

### Mass spectrometry

Immunoprecipitates were washed with 100mM NH_4_HCO_3_ and reduced (10 mM DTT, 56°C for 45 min) and alkylated (50 mM iodoacetamide, in the dark at room temperature for 1 h). Proteins were subsequently digested with trypsin (sequencing grade, Promega, Madison, WI) at an enzyme/substrate ratio of 1:50 at room temperature overnight in 100 mM NH_4_HCO_3_ pH8. Trypsin activity was quenched by adding formic acid to a final concentration of 5%. Peptides were desalted using C18 SpinTips (Protea, Morgantown, WV) then vacuum centrifuged to near dryness and stored at −80°C. Peptide labeling with TMT 6plex (Thermo Fisher Scientific) was performed per manufacturer’s instructions. Samples were dissolved in 70 μL ethanol and 30 μL of 500 mM triethylammonium bicarbonate, pH8.5, and the TMT reagent was dissolved in 30 μL of anhydrous acetonitrile. The solution containing peptides and TMT reagent was vortexed and incubated at room temperature for 1 h. Samples labeled with the six different isobaric TMT reagents were combined and concentrated to completion in a vacuum centrifuge. The peptides were separated by reverse phase HPLC using an EASY- nLC1000 system (Thermo Fisher Scientific) over a 140-min gradient followed by nanoelectrospray using a QExactive mass spectrometer (Thermo Fisher Scientific). The mass spectrometer was operated in a data-dependent mode. The parameters for the full scan MS were: resolution of 70,000 across 350–2000 *m/z*, AGC 3e^6^, and maximum IT 50 ms. The full MS scan was followed by MS/MS for the top 10 precursor ions in each cycle with a NCE of 32 and dynamic exclusion of 30 s. Raw mass spectral data files (.raw) were searched using Proteome Discoverer (Thermo Fisher Scientific) and Mascot version 2.4.1 (Matrix Science). Mascot search parameters were: 10 ppm mass tolerance for precursor ions; 10mmu for fragment ion mass tolerance; 2 missed cleavages of trypsin. Fixed modifications were carbamidomethylation of cysteine and TMT 6plex modification of lysines and peptide N-termini; variable modification was oxidized methionine. Only peptides with a Mascot score greater than or equal to 25 and an isolation interference less than or equal to 30 were included in the quantitative data analysis. TMT quantification was obtained using Proteome Discoverer and isotopically corrected per manufacturer’s instructions. Mass spectrometry proteomics data have been deposited to the ProteomeXchange Consortium (http://proteomecentral.proteomexchange.org) via the PRIDE partner repository [56] with the dataset identifier PXD005473.

### RNA immunoprecipitation and sequencing (RIP-seq)/qPCR

MEIOC RIP-seq and IgG RIP-seq were carried out on P15 testes from wild-type C57BL/6 male mice (N = 2 per RIP-seq type). MEIOC RIP-seq was also carried out on P15 testes from wild-type and *Meioc-*deficient littermates (N = 2 per genotype). YTHDC2 RIP-seq and IgG RIP-seq were carried out on P20 testes from wild-type C57BL/6 male mice (N = 2 per RIP-seq type). To prepare lysates, testis pairs were isolated and lysed under non-reducing conditions (50mM Tris-HCl, pH7.4, 100mM NaCl, 1% NP-40, 0.1% SDS, 0.5% sodium deoxycholate) supplemented with 40U/mL RNAseOUT (Thermo Fisher Scientific) and EDTA-free protease inhibitor (Roche Diagnostics). Lysates were incubated at 4°C with rotation for 15–25 min and cleared using Ultrafiltration Spin Columns, 0.45 μm cutoff (EMD Millipore). Dynabeads Protein G were washed twice with lysis buffer and resuspended in lysis buffer at the original volume. The soluble lysate from each testis pair was pre-cleared for 1 h at 4°C with 100 μl of Dynabeads Protein G (Thermo Fisher Scientific), and 40–80 μL was set aside as the input control. Beads were prepared by incubating 5 μg of anti-MEIOC antibody (antibody generation described above), one of two anti-YTHDC2 antibodies (Santa Cruz Biotechnology sc-249370 or Bethyl Laboratories A303-026A), or normal rabbit or goat IgG (Santa Cruz Biotechnology) per 100 μL Dynabeads with rotation for 45–60 min at room temperature. For immunoprecipitation, 570 μL lysate was incubated with 100 μL antibody-bound Dynabeads with rotation for 2 h at 4°C. The beads were then washed six times for 5 min with rotation in wash buffer (50mM Tris-HCl, pH7.4, 300mM NaCl, 1mM EDTA, 1% NP-40, 0.1% SDS, and 0.5% sodium deoxycholate). A subset of the immunoprecipitate was then set aside for immunoblotting to verify successful immunoprecipitation of MEIOC and YTHDC2 (immunoblotting described above). The RNA from immunoprecipitates and input control was released by adding an additional 0.125% SDS and 250 mg/mL Proteinase K (Thermo Fisher Scientific) and incubating for 30 min with shaking at 37˚C. RNA was isolated via extraction with acid phenol:chloroform:IAA, pH4.5 (Thermo Fisher Scientific) using phase lock gel tubes (5 PRIME) according to the manufacturer’s protocol. Extracted RNA was supplemented with GlycoBlue (Thermo Fisher Scientific) to 37.5 μg/mL and sodium acetate, pH5.5, to 0.1M. RNA was precipitated overnight at -20˚C in two volumes of 100% ethanol, pelleted by spinning for 20 min at 16,000 *g* at room temperature, washed once with 80% ethanol, dried, and resuspended in 25 μl water. For each sample, 5 μL of RNA was kept for qPCR analysis and the remaining RNA was used for sequencing library preparation via the SMARTer Stranded RNA-Seq Kit (ClonTech). Libraries from each RNA immunoprecipitation experiment (MEIOC or YTHDC2 RIP, IgG control RIP, and input control) were multiplexed and sequenced on the Illumina MiSeq platform. MEIOC RIP libraries were sequenced with 52-base-pair single-end reads. YTHDC2 RIP libraries were sequenced with 26-base-pair paired-end reads. Sequencing data have been deposited in NCBI GEO under accession number GSE90702 and NCBI SRA under accession number SRP094112. For qPCR analysis, RNA was reverse transcribed using Superscript VILO Master Mix (Thermo Fisher Scientific) and analyzed in triplicate using Power SYBR Green PCR Master Mix (Thermo Fisher Scientific) according to the manufacturer’s protocol on a 7500 Fast Real-Time PCR System (Applied Biosystems). Primers for qPCR analyses are listed in S8 Table. Results were analyzed using *Actb* expression as a non-target normalization control and calculating the fold change over the IgG control RIP.

### DESeq analysis of RIP-seq data

Prior to mapping, reads were trimmed for a minimum quality score of 20 and the first three bases of the first sequencing read, which were added during SMARTer Stranded library preparation, were removed using Cutadapt v1.8. Reads were aligned to the mouse genome (mm10) via TopHat v2.0.13 using default parameters and supplying the RefSeq transcript annotation. Alignments were converted to counts using HTSeq v0.6.1p1, using the “–a” option to skip reads whose alignment quality indicated non-unique alignments (i.e., alignment quality <50). DESeq2 v1.10.1 was then used to estimate RIP-seq enrichments resulting from MEIOC or YTHDC2 binding. DESeq2’s default procedure was applied to normalize read counts across all samples. Data were analyzed with multi-factor designs to estimate protein-specific binding over controls. For YTHDC2 RIP-seq data, log2(read counts) for each gene was modeled as a linear combination of the gene-specific effects of three variables: binding to YTHDC2 (“YTHDC2”), binding to IgG (“IgG”), and batch (“batch”) (S9A Table). The last variable captured differences due to the YTHDC2 antibody used and sequencing batch. This model identified transcripts that were enriched in YTHDC2 RIP-seq datasets generated using both antibodies. MEIOC analyses included RIP-seq experiments performed on wild-type and knockout samples. Read-count differences between wild-type and *Meioc*-deficient RIP-seq samples thus reflect both the effects of MEIOC protein binding and gene expression differences due to the *Meioc* genotype. To estimate the former independently of the latter, wild-type and *Meioc*-deficient RIP-seq and RNA-seq data were analyzed jointly, modeling log2(read counts) as a linear combination of five variables: genotype, binding to MEIOC protein (“Meioc.specific”), binding to MEIOC antibody (“Meioc.nonspecific”), binding to IgG antibody (“IgG”), and sequencing batch (S9B Table). (For this analysis, RNA-seq data were summarized as gene-level read counts obtained from HTSeq, processed identically to the RIP-seq samples.) Enrichments (FDR < 0.05; fold change > 3 for MEIOC; fold change > 2 for YTHDC2) are reported as the fold changes between samples with and without protein-specific binding, independent of the effects of non-specific binding and sequencing batch. These were obtained from the *results* function in DESeq2 supplying the argument: *contrast = c(“YTHDC2”*, *1*, *0) or contrast = c(“Meioc*.*specific”*, *1*, *0)*. For RIP-associated RNA-seq data, FPKMs were obtained using Cuffnorm v2.2.1 [54].

## Supporting information

S1 Fig MEIOC is conserved in vertebrates. Alignment of electronic predictions of MEIOC orthologs. We searched for homologs of mouse MEIOC (NP_001121048.1) by querying the RefSeq protein database by blastp, and the translated NCBI nucleotide collection database by tblastn. Both methods yielded similar results. We restricted the search to the following representative species: *Mus musculus*, *Rattus norvegicus*, *Canis familiaris*, *Monodelphis domestica*, *Homo sapiens*, *Pan troglodytes*, *Anolis carolinensis*, G*allus gallus*, *Xenopus tropicalis*, *Danio rerio*, *Branchiostoma floridae*, *Ciona intestinalis*, *Strongylocentrotus purpuratus*, *Bombyx mori*, *Caenorhabditis elegans*, *Nematostella vectensis*, *Petromyzon marinus*, *Drosophila melanogaster*, *Saccharomyces cerevisiae*. Homologs of mouse MEIOC (>80% query coverage and >30% identity) were aligned by Clustal Omega and visualized by Jalview (shown in figure). The box denotes a conserved domain annotated by PFAM as PF15189. Additional matches to MEIOC, restricted to the region corresponding to PF15189, were found in *Danio rerio*, *Branchiostoma floridae*, *Ciona intestinalis*, *Strongylocentrotus purpuratus*, *Bombyx mori*, *Caenorhabditis elegans*, and *Nematostella vectensis*. We were unable to identify matches to either full-length mouse MEIOC or the region corresponding to PF15189 in *Petromyzon marinus*, *Drosophila melanogaster*, and *Saccharomyces cerevisiae*.
(TIF)

S2 Fig Testicular expression of *Meioc* is conserved. Expression of *Meioc* homologs in tissues from human, mouse, rat, and chicken as measured by RNAseq. RNAseq data of tissue panel from various species from Merkin et al., 2012. Expression of *Meioc* is measured in fragments per kilobase per million reads (FPKM). Amongst the species and tissues sampled, *Meioc* expression is predominantly in the testis. ND = no data. Note that the chromosomal events of meiotic prophase occur in the female during fetal stages, so we do not necessarily expect *Meioc* expression in the adult ovary.
(TIF)

S3 Fig Immunofluorescence for MEIOC in wild-type and *Meioc*-deficient E16.5 ovary and P15 testis. Rabbit anti-MEIOC antibodies were generated to a peptide corresponding to the terminal 20 amino acids of mouse MEIOC (CHESINSSNPMNQRGETSKH). Germ cell-specific staining was observed in wild-type ovary and testis, but was absent in *Meioc*-/- ovary and testis. Sections were co-stained for MVH, to identify germ cells, and with DAPI to mark nuclei.
(TIF)

S4 Fig Generation of *Meioc* mutant alleles. (A) The *Meioc* gene was targeted for homologous recombination with a targeting vector for a knockout-first allele of *Meioc* (obtained from the KOMP Repository, vector PG00048_X_6_E03). Briefly, a 0.8 kb region containing exon 3 of the *Meioc* gene was replaced with a lacZ reporter, Neo selection marker, and exon 3, flanked by FRT (green triangles) and loxP (red triangles) sites. K: KpnI restriction site; X: XhoI restriction site. a, b, c, d, e: genotyping primers described in (F, G).
(B) The homologously targeted allele, denoted 3lox as it retains 3 loxP sites. The homologously targeted allele yields a 10.8 kb K/X fragment, whereas the wild-type allele yields a 18.9 kb K/X fragment. In the 3lox allele, *Meioc* is expected to be disrupted by the active lacZ reporter.
(C) Conversion of the 3lox allele to a conditional allele, denoted 2lox, by Flp recombination. The lacZ and Neo genes are excised, leaving exon 3 flanked by loxP sites.
(D) Conversion of the 2lox allele to a knockout allele, denoted 1lox, or *Meioc*-, by Cre recombination. Exon 3 of Meioc is excised. Both *Meioc* 3lox/3lox and *Meioc* 1lox/1lox (*Meioc* -/-) mice are considered *Meioc*-deficient.
(E) Southern blot confirmation of correctly targeted ES cell clones using a KpnI/XhoI restriction digest, and a probe 3’ of the 3’ homology arm.
(F) PCR assays for genotyping of wild-type (+/+), 3lox (3L), 2lox (2L), and 1lox (1L or -) alleles.
(G) Germline transmission of various *Meioc* alleles verified using indicated PCR assays.
(TIF)

S5 Fig Histological analyses of *Meioc*-deficient adult testis and ovary. (A) Wild-type and *Meioc* 3L/3L P30 testis and ovary.
(B, C) Hematoxylin and eosin-stained sections of adult (>8 weeks) testes from (B) wild-type and *Meioc* 3L/3L mice and (C) wild-type and *Meioc* -/- male mice. *Meioc*-deficient testes completely lacked postmeiotic germ cells, and were depleted for meiotic germ cells. The extent of this depletion varied among mice of mixed background: in some individuals, germ cells did not progress past preleptotene (prior to meiotic prophase), while in others, germ cells advanced to the zygotene stage of meiotic prophase. To obtain a reproducible phenotype, we backcrossed the *Meioc* mutant alleles onto the C57BL/6 background. In backcrossed mice, we consistently found that germ cells advanced to the zygotene stage. All experiments reported in the main text were performed in mice backcrossed to the C57BL/6 background between five to seven generations (96.9–99.2% of genome expected to be of C57BL/6 origin), unless otherwise noted. All results were obtained using both *Meioc* 3L/3L and *Meioc* -/- mice, and phenotypes were consistent between the two alleles. pL–preleptotene spermatocyte, L–leptotene spermatocyte, Z–zygotene spermatocyte, P–pachytene spermatocyte, D–diplotene spermatocyte, ML–metaphase-like, rSt–round spermatid, St–spermatid, spz–spermatozoa.
(D) Hematoxylin and eosin-stained sections of adult ovaries from wild-type and *Meioc* -/- female mice. Wild-type adult ovaries contain oocytes contained within follicles at various stages of maturation (arrowheads). *Meioc* -/- adult ovaries are devoid of oocytes.
(TIFF)

S6 Fig TUNEL analyses of *Meioc*-deficient adult testis and ovary. (A) Wild-type and 1L/1L adult testis. In *Meioc*-/- adult testis, TUNEL staining was readily detected in cells with condensed (C) or apoptotic (A) nuclei. TUNEL staining was not detected in preleptotenes (pL) or in cells with metaphase-like chromosome condensation (M). TUNEL-positive cells were rarely detected in wild-type adult testes. Scale bar = 10 μm.
(B) Wild-type and 1L/1L E14.5 ovary. Low magnification images: most cells in both wild-type and *Meioc*-/- ovaries (o) were TUNEL-negative. High magnification image: a few TUNEL-positive cells were detected in the wild-type ovary. m, mesonephros. Scale bar = 10 μm (low magnification images) or 3.3 μm (high magnification image).
(TIF)

S7 Fig Quantification of tubules containing metaphase-like cells. Percentage of tubule cross-sections containing preleptotene (pL), leptotene (L), zygotene (Z) and pachytene (P) cells in P15 *Meioc* -/- and control testes. We also determined the percentage of tubules containing metaphase-like cells, cells with condensed nuclei, or apoptotic cells. When a tubule contained, for example, a metaphase-like cell, we noted the stage of meiotic prophase found in that tubule. Each vertical column represents counts from one animal.
(TIFF)

S8 Fig *Meioc*-deficient testicular and ovarian germ cells express molecular markers of meiotic prophase. (A) Immunofluorescence staining for DMC1, γH2AX, and SYCP3 in chromosome spreads from wild-type and *Meioc* -/- germ cells from E16.5 ovaries. DNA stained by DAPI. In wild-type germ cells, we observed DMC1, γH2AX, and SYCP3 localization consistent with leptotene, and zygotene stages of meiotic prophase. In *Meioc* -/- germ cells, the most advanced stage of meiotic prophase we observed was leptotene stage. Although metaphase-like cells were observed in histological sections, we were unable to identify any metaphase-like cells in spreads.
(B) Frequencies of leptotene, zygotene, pachytene, or metaphase-like germ cells, or germ cells with other abnormal morphology, in cell spreads from P15 *Meioc* -/- and wild-type testes.
(TIFF)

S9 Fig *Meioc*-deficient testicular and ovarian germ cells express molecular markers of metaphase. Immunofluorescence staining for LAMIN and pH3 in wild-type and *Meioc*-/- P15 testis and E16.5 ovary sections. Nuclei are stained by DAPI. In wild-type P15 testis and E16.5 ovary, meiotic germ cell nuclei are still intact, as detected by LAMIN staining, and no pH3 is observed. In *Meioc*-/- P15 testis and E16.5 ovary, LAMIN is not detected in germ cells which have condensed their nuclei and are pH3+. LAMIN and pH3 staining of wild-type adult testicular germ cells in metaphase I are shown for comparison.
(TIF)

S10 Fig YTHDC2 is conserved in verterbrates. Alignment of electronic predictions of YTHDC2 orthologs. We searched for homologs of mouse YTHDC2 (NP_001156485) by querying the RefSeq protein database by blastp. We restricted the search to the following representative species: *Mus musculus*, *Rattus norvegicus*, *Canis familiaris*, *Monodelphis domestica*, *Homo sapiens*, *Pan troglodytes*, *Anolis carolinensis*, G*allus gallus*, *Xenopus tropicalis*, *Danio rerio*, *Branchiostoma floridae*, *Ciona intestinalis*, *Strongylocentrotus purpuratus*, *Bombyx mori*, *Caenorhabditis elegans*, *Nematostella vectensis*, *Petromyzon marinus*, *Drosophila melanogaster*, *Saccharomyces cerevisiae*. Homologs of mouse YTHDC2 (≥75% query coverage and ≥25% identity) were aligned by Clustal Omega and visualized by Jalview (shown in figure). In *Drosophila melanogaster*, the homolog was annotated as benign gonial cell neoplasm (BGCN; NP_523832.2). Additional matches to YTHDC2 were found in *Danio rerio*, *Branchiostoma floridae*, *Ciona intestinalis*, *Strongylocentrotus purpuratus*, *Bombyx mori*, *Caenorhabditis elegans*, and *Nematostella vectensis*. We were unable to identify matches to full-length mouse YTHDC2 in *Petromyzon marinus* and *Saccharomyces cerevisiae*.
(TIF)

S11 Fig MEIOC and IgG RIP-qPCR. A subset of targets and non-targets, identified via RIP-Seq and enrichment analysis, were verified via qPCR of the same P15 RIP samples analyzed via sequencing (N = 2). All ΔΔCt values were normalized to *Actb* qPCR results and displayed as fold change over IgG RIP-qPCR. Error bars represent s.e.m. Overall trends of target abundance in MEIOC RIP compared to IgG RIP are consistent with RIP-seq results. However, statistical analysis (one-tailed, paired Student t-test) did not show the statistical enrichment of any target in the MEIOC RIP (p>0.05 for all targets).
(TIF)

S1 Table Gene expression levels and fold changes of wild-type and *Meioc* -/- E14.5 ovaries. (XLSX)

S2 Table GO categories enriched in genes expressed at higher or lower levels in E14.5 *Meioc* -/- ovaries. (XLSX)

S3 Table Unique peptides enriched in MEIOC immunoprecipitation (IP), identified via quantitative mass spectrometry. Samples A are IgG IP from wild-type lysates; samples B are MEIOC IP from wild-type lysates; and samples C are MEIOC IP from *Meioc*-deficient lysates.
(XLSX)

S4 Table Enrichment in MEIOC RIP-seq and YTHDC2 RIP-seq from postnatal testis. MEIOC targets were defined as exhibiting a fold change >3, FDR<0.05, and FPKM>1; YTHDC2 targets were defined as exhibiting a fold change >2, FDR<0.05, and FPKM>1.
(XLSX)

S5 Table MEIOC and YTHDC2 targets from postnatal testis that are differentially expressed in *Meioc*-deficient and wild-type E14.5 ovaries. MEIOC and YTHDC2 targets were identified from the RIP-seq analysis.
(XLSX)

S6 Table Antibodies and experimental conditions for immunofluorescence stainings performed in this study. (DOCX)

S7 Table Single molecule FISH probes used in this study. (XLSX)

S8 Table List of qPCR primers used in this study. (DOCX)

S9 Table Coding of sample types for DESeq analysis of YTHDC2 and MEIOC RIP. (DOCX)
