# Supplementary figures and images for: Meioc maintains an extended meiotic prophase I in mice
Source: PLoS Genet. 2017 Apr 5;13(4):e1006704. doi: 10.1371/journal.pgen.1006704 (PMC5397071; doi:10.1371/journal.pgen.1006704)

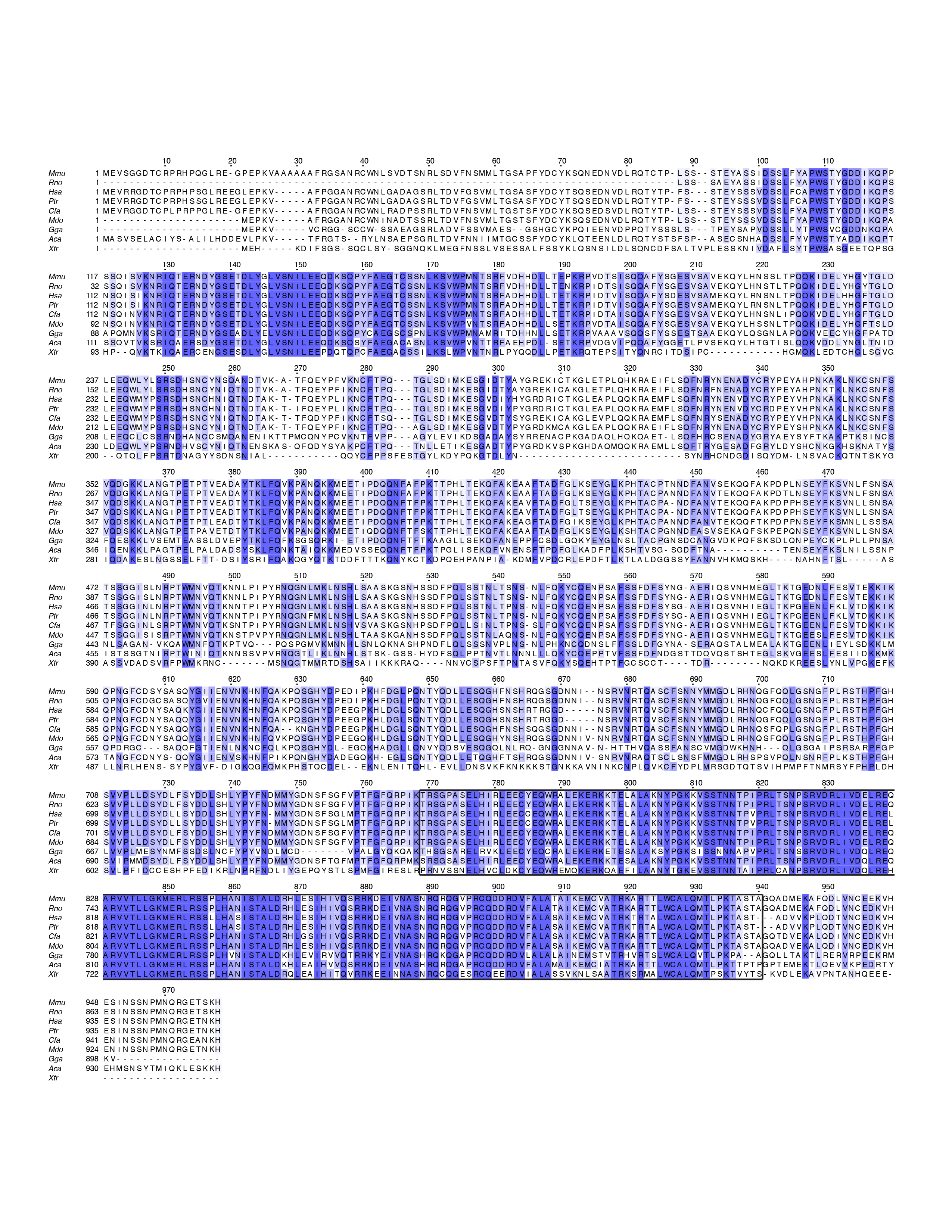

Supplement: S1 Fig — Alignment of electronic predictions of MEIOC orthologs. We searched for homologs of mouse MEIOC (NP_001121048.1) by querying the RefSeq protein database by blastp, and the translated NCBI nucleotide collection database by tblastn. Both methods yielded similar results. We restricted the search to the following representative species: Mus musculus, Rattus norvegicus, Canis familiaris, Monodelphis domestica, Homo sapiens, Pan troglodytes, Anolis carolinensis, Gallus gallus, Xenopus tropicalis, Danio rerio, Branchiostoma floridae, Ciona intestinalis, Strongylocentrotus purpuratus, Bombyx mori, Caenorhabditis elegans, Nematostella vectensis, Petromyzon marinus, Drosophila melanogaster, Saccharomyces cerevisiae. Homologs of mouse MEIOC (>80% query coverage and >30% identity) were aligned by Clustal Omega and visualized by Jalview (shown in figure). The box denotes a conserved domain annotated by PFAM as PF15189. Additional matches to MEIOC, restricted to the region corresponding to PF15189, were found in Danio rerio, Branchiostoma floridae, Ciona intestinalis, Strongylocentrotus purpuratus, Bombyx mori, Caenorhabditis elegans, and Nematostella vectensis. We were unable to identify matches to either full-length mouse MEIOC or the region corresponding to PF15189 in Petromyzon marinus, Drosophila melanogaster, and Saccharomyces cerevisiae. (TIF) [file pgen.1006704.s001.tif]

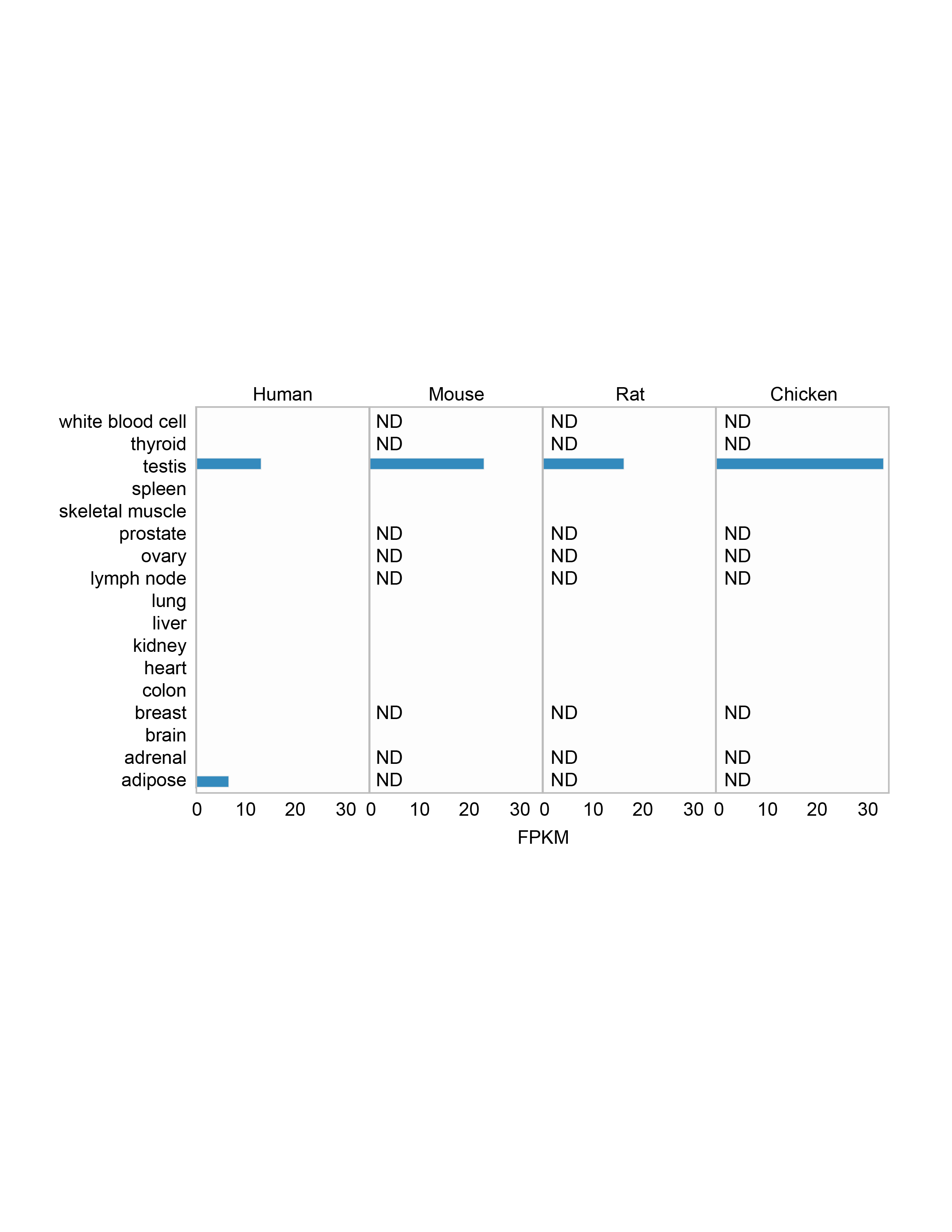

Supplement: S2 Fig — Expression of Meioc homologs in tissues from human, mouse, rat, and chicken as measured by RNAseq. RNAseq data of tissue panel from various species from Merkin et al., 2012. Expression of Meioc is measured in fragments per kilobase per million reads (FPKM). Amongst the species and tissues sampled, Meioc expression is predominantly in the testis. ND = no data. Note that the chromosomal events of meiotic prophase occur in the female during fetal stages, so we do not necessarily expect Meioc expression in the adult ovary. (TIF) [file pgen.1006704.s002.tif]

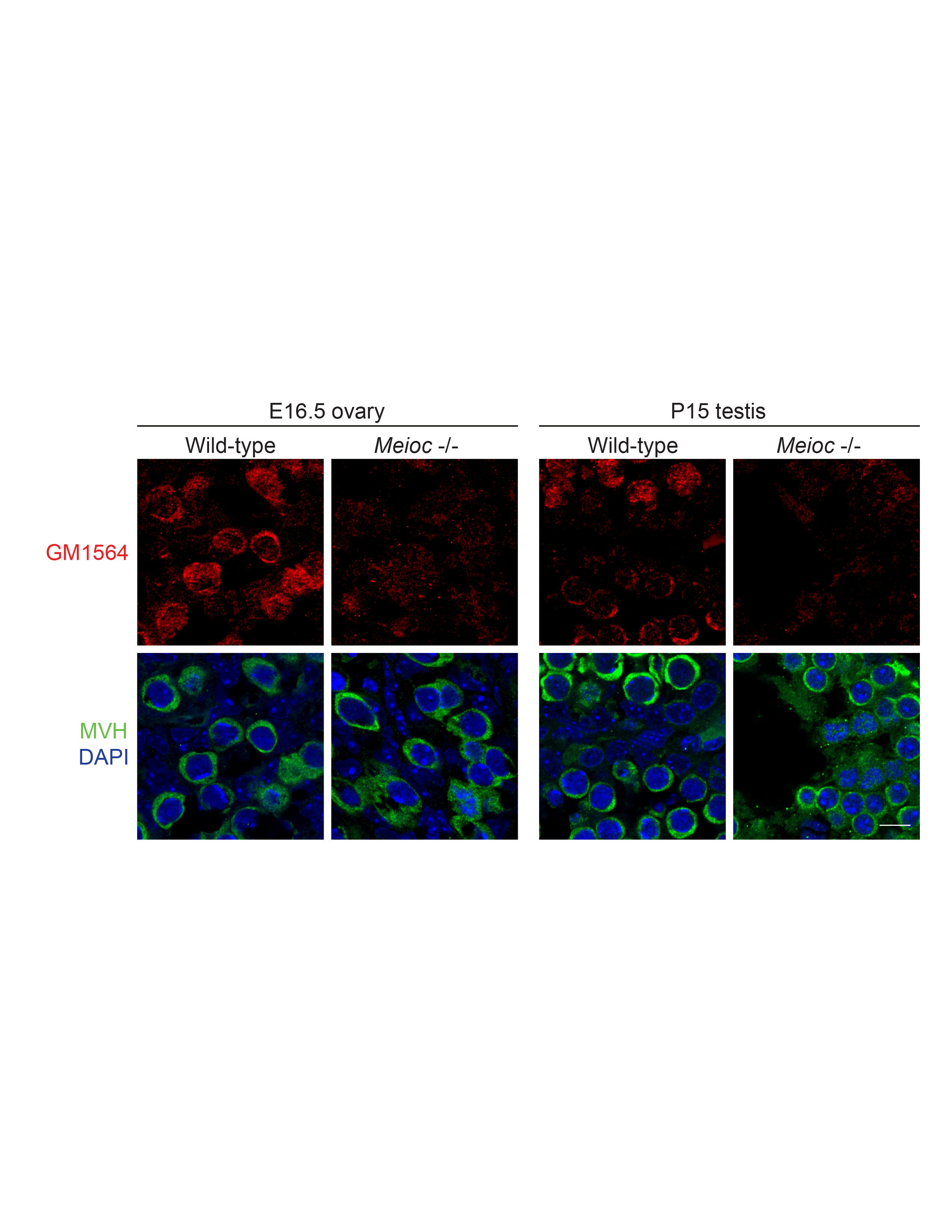

Supplement: S3 Fig — Rabbit anti-MEIOC antibodies were generated to a peptide corresponding to the terminal 20 amino acids of mouse MEIOC (CHESINSSNPMNQRGETSKH). Germ cell-specific staining was observed in wild-type ovary and testis, but was absent in Meioc-/- ovary and testis. Sections were co-stained for MVH, to identify germ cells, and with DAPI to mark nuclei. (TIF) [file pgen.1006704.s003.tif]

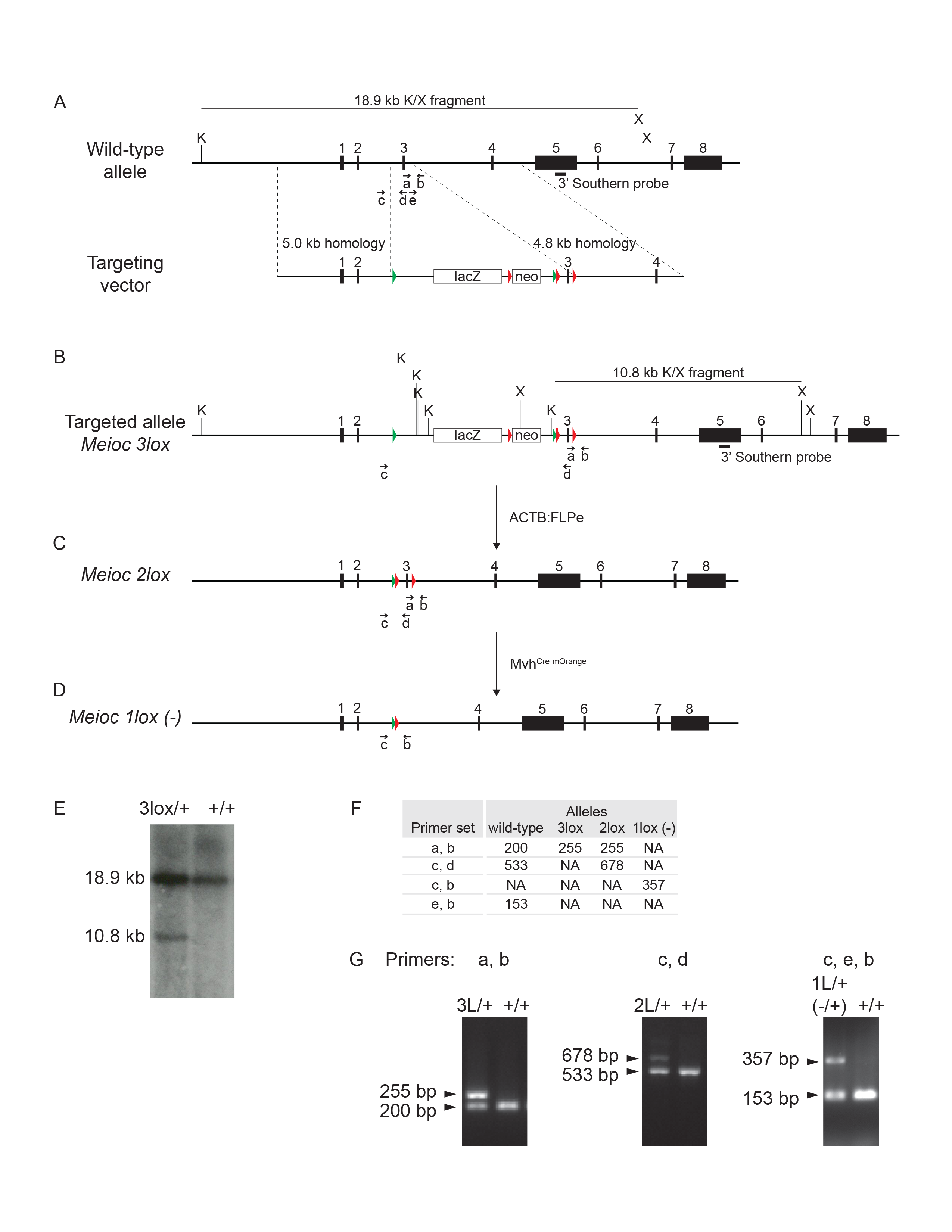

Supplement: S4 Fig — (A) The Meioc gene was targeted for homologous recombination with a targeting vector for a knockout-first allele of Meioc (obtained from the KOMP Repository, vector PG00048_X_6_E03). Briefly, a 0.8 kb region containing exon 3 of the Meioc gene was replaced with a lacZ reporter, Neo selection marker, and exon 3, flanked by FRT (green triangles) and loxP (red triangles) sites. K: KpnI restriction site; X: XhoI restriction site. a, b, c, d, e: genotyping primers described in (F, G). (B) The homologously targeted allele, denoted 3lox as it retains 3 loxP sites. The homologously targeted allele yields a 10.8 kb K/X fragment, whereas the wild-type allele yields a 18.9 kb K/X fragment. In the 3lox allele, Meioc is expected to be disrupted by the active lacZ reporter. (C) Conversion of the 3lox allele to a conditional allele, denoted 2lox, by Flp recombination. The lacZ and Neo genes are excised, leaving exon 3 flanked by loxP sites. (D) Conversion of the 2lox allele to a knockout allele, denoted 1lox, or Meioc-, by Cre recombination. Exon 3 of Meioc is excised. Both Meioc 3lox/3lox and Meioc 1lox/1lox (Meioc -/-) mice are considered Meioc-deficient. (E) Southern blot confirmation of correctly targeted ES cell clones using a KpnI/XhoI restriction digest, and a probe 3’ of the 3’ homology arm. (F) PCR assays for genotyping of wild-type (+/+), 3lox (3L), 2lox (2L), and 1lox (1L or -) alleles. (G) Germline transmission of various Meioc alleles verified using indicated PCR assays. (TIF) [file pgen.1006704.s004.tif]

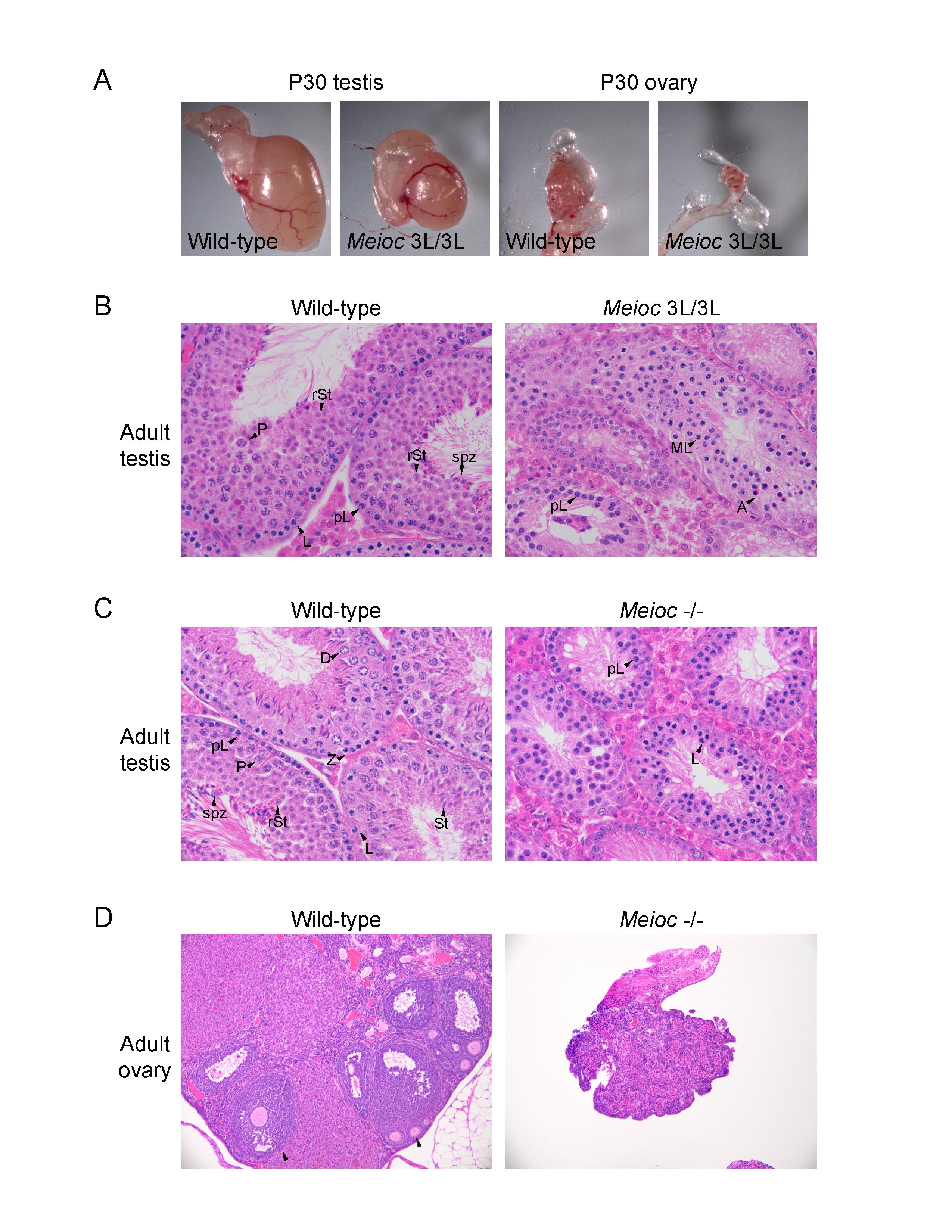

Supplement: S5 Fig — (A) Wild-type and Meioc 3L/3L P30 testis and ovary. (B, C) Hematoxylin and eosin-stained sections of adult (>8 weeks) testes from (B) wild-type and Meioc 3L/3L mice and (C) wild-type and Meioc -/- male mice. Meioc-deficient testes completely lacked postmeiotic germ cells, and were depleted for meiotic germ cells. The extent of this depletion varied among mice of mixed background: in some individuals, germ cells did not progress past preleptotene (prior to meiotic prophase), while in others, germ cells advanced to the zygotene stage of meiotic prophase. To obtain a reproducible phenotype, we backcrossed the Meioc mutant alleles onto the C57BL/6 background. In backcrossed mice, we consistently found that germ cells advanced to the zygotene stage. All experiments reported in the main text were performed in mice backcrossed to the C57BL/6 background between five to seven generations (96.9–99.2% of genome expected to be of C57BL/6 origin), unless otherwise noted. All results were obtained using both Meioc 3L/3L and Meioc -/- mice, and phenotypes were consistent between the two alleles. pL–preleptotene spermatocyte, L–leptotene spermatocyte, Z–zygotene spermatocyte, P–pachytene spermatocyte, D–diplotene spermatocyte, ML–metaphase-like, rSt–round spermatid, St–spermatid, spz–spermatozoa. (D) Hematoxylin and eosin-stained sections of adult ovaries from wild-type and Meioc -/- female mice. Wild-type adult ovaries contain oocytes contained within follicles at various stages of maturation (arrowheads). Meioc -/- adult ovaries are devoid of oocytes. (TIFF) [file pgen.1006704.s005.tiff]

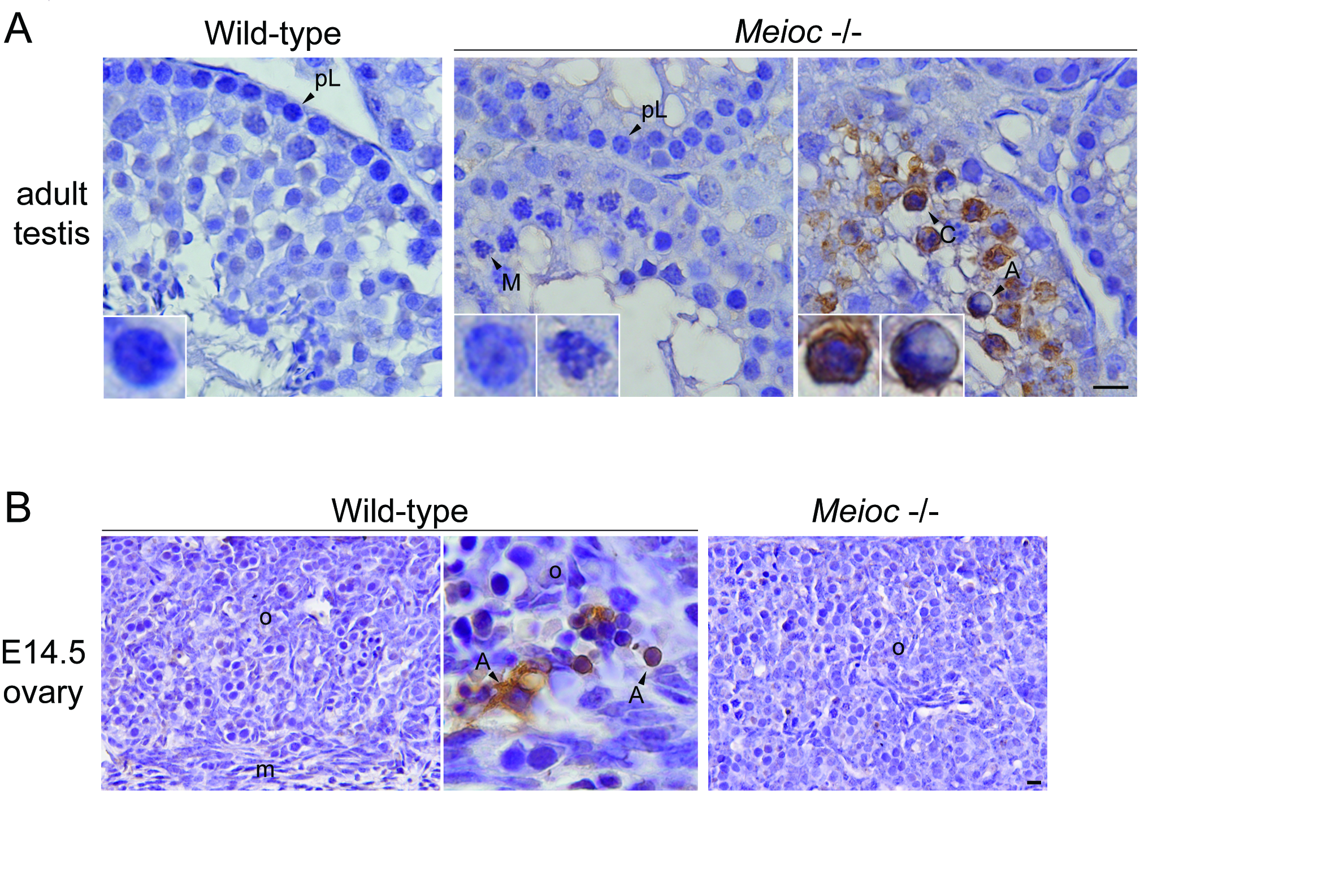

Supplement: S6 Fig — (A) Wild-type and 1L/1L adult testis. In Meioc-/- adult testis, TUNEL staining was readily detected in cells with condensed (C) or apoptotic (A) nuclei. TUNEL staining was not detected in preleptotenes (pL) or in cells with metaphase-like chromosome condensation (M). TUNEL-positive cells were rarely detected in wild-type adult testes. Scale bar = 10 μm. (B) Wild-type and 1L/1L E14.5 ovary. Low magnification images: most cells in both wild-type and Meioc-/- ovaries (o) were TUNEL-negative. High magnification image: a few TUNEL-positive cells were detected in the wild-type ovary. m, mesonephros. Scale bar = 10 μm (low magnification images) or 3.3 μm (high magnification image). (TIF) [file pgen.1006704.s006.tif]

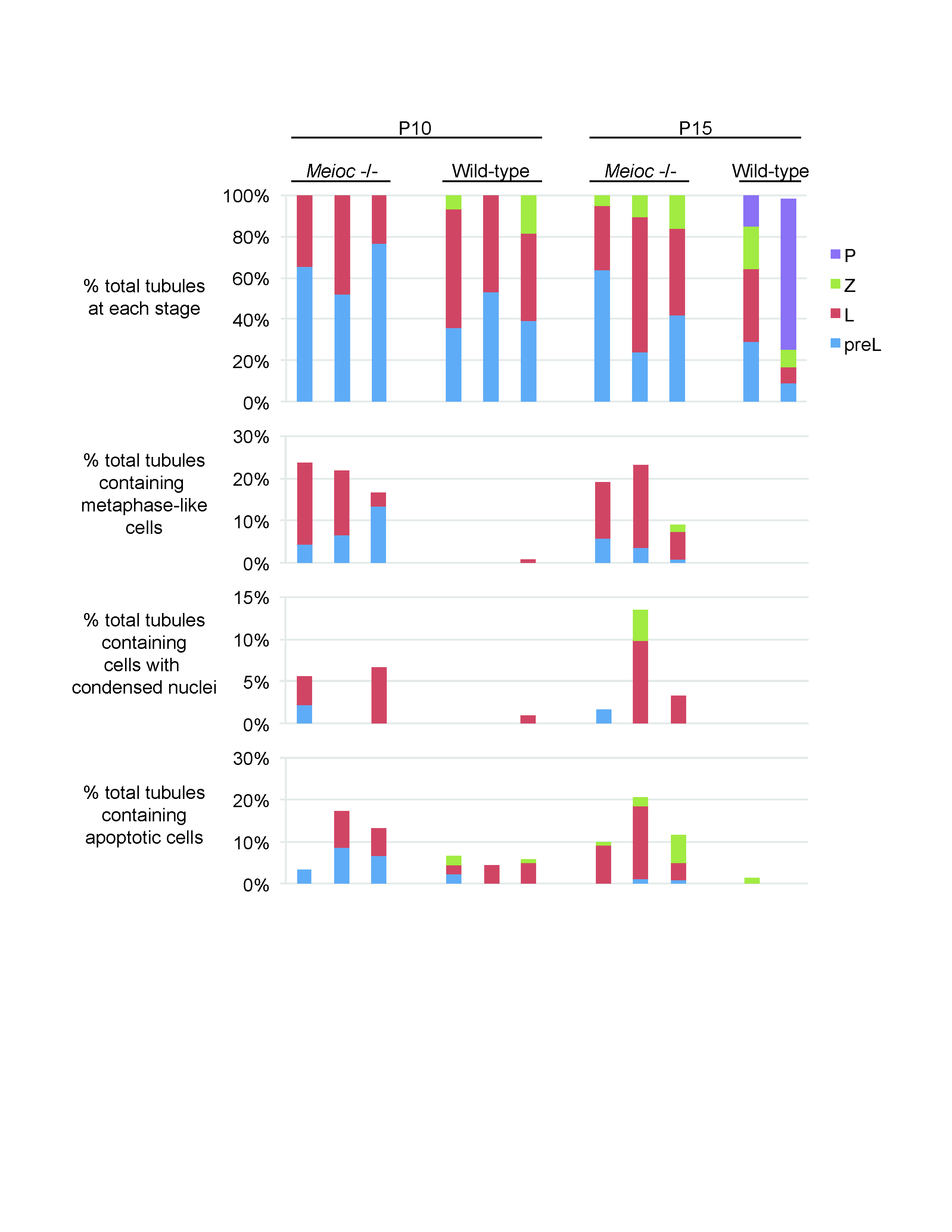

Supplement: S7 Fig — Percentage of tubule cross-sections containing preleptotene (pL), leptotene (L), zygotene (Z) and pachytene (P) cells in P15 Meioc -/- and control testes. We also determined the percentage of tubules containing metaphase-like cells, cells with condensed nuclei, or apoptotic cells. When a tubule contained, for example, a metaphase-like cell, we noted the stage of meiotic prophase found in that tubule. Each vertical column represents counts from one animal. (TIFF) [file pgen.1006704.s007.tiff]

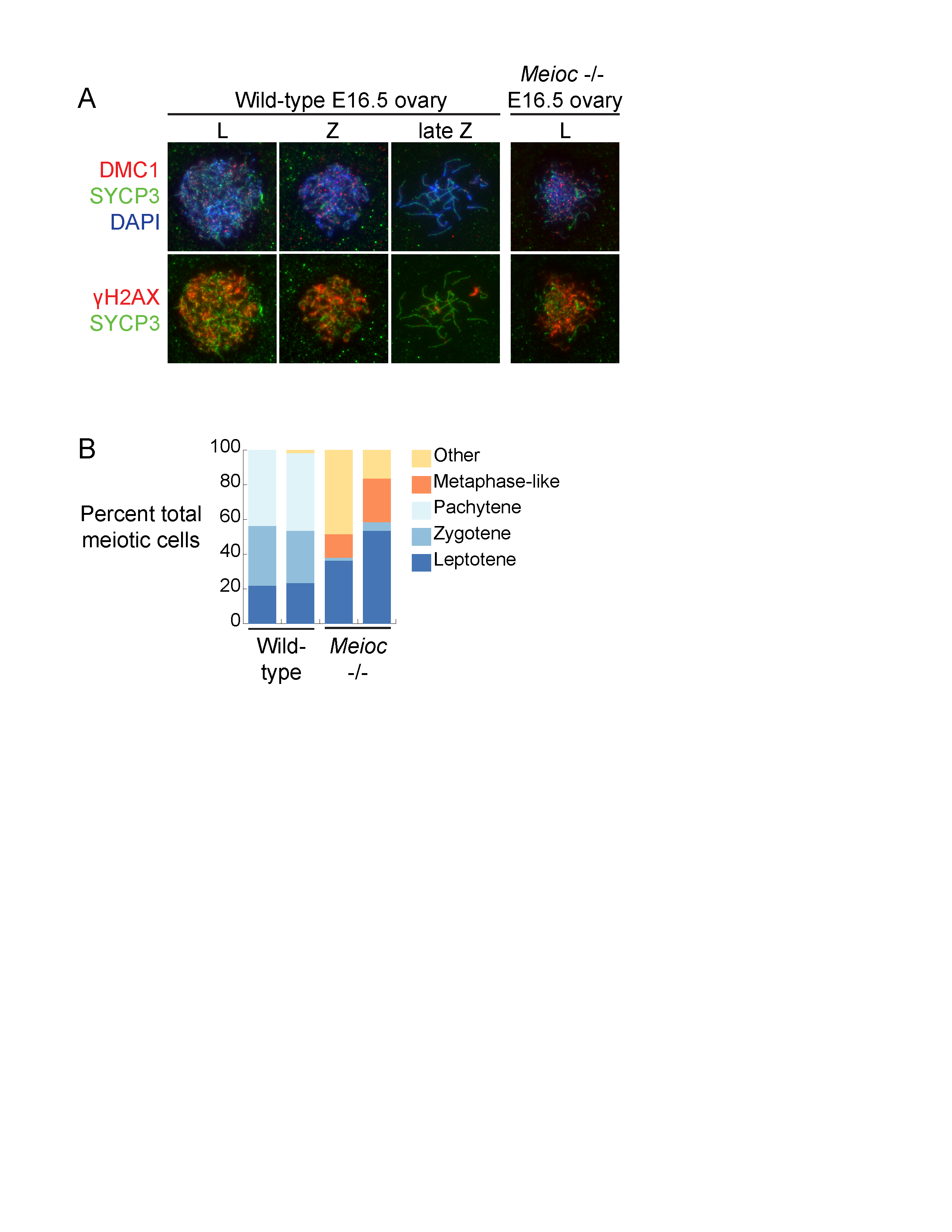

Supplement: S8 Fig — (A) Immunofluorescence staining for DMC1, γH2AX, and SYCP3 in chromosome spreads from wild-type and Meioc -/- germ cells from E16.5 ovaries. DNA stained by DAPI. In wild-type germ cells, we observed DMC1, γH2AX, and SYCP3 localization consistent with leptotene, and zygotene stages of meiotic prophase. In Meioc -/- germ cells, the most advanced stage of meiotic prophase we observed was leptotene stage. Although metaphase-like cells were observed in histological sections, we were unable to identify any metaphase-like cells in spreads. (B) Frequencies of leptotene, zygotene, pachytene, or metaphase-like germ cells, or germ cells with other abnormal morphology, in cell spreads from P15 Meioc -/- and wild-type testes. (TIFF) [file pgen.1006704.s008.tiff]

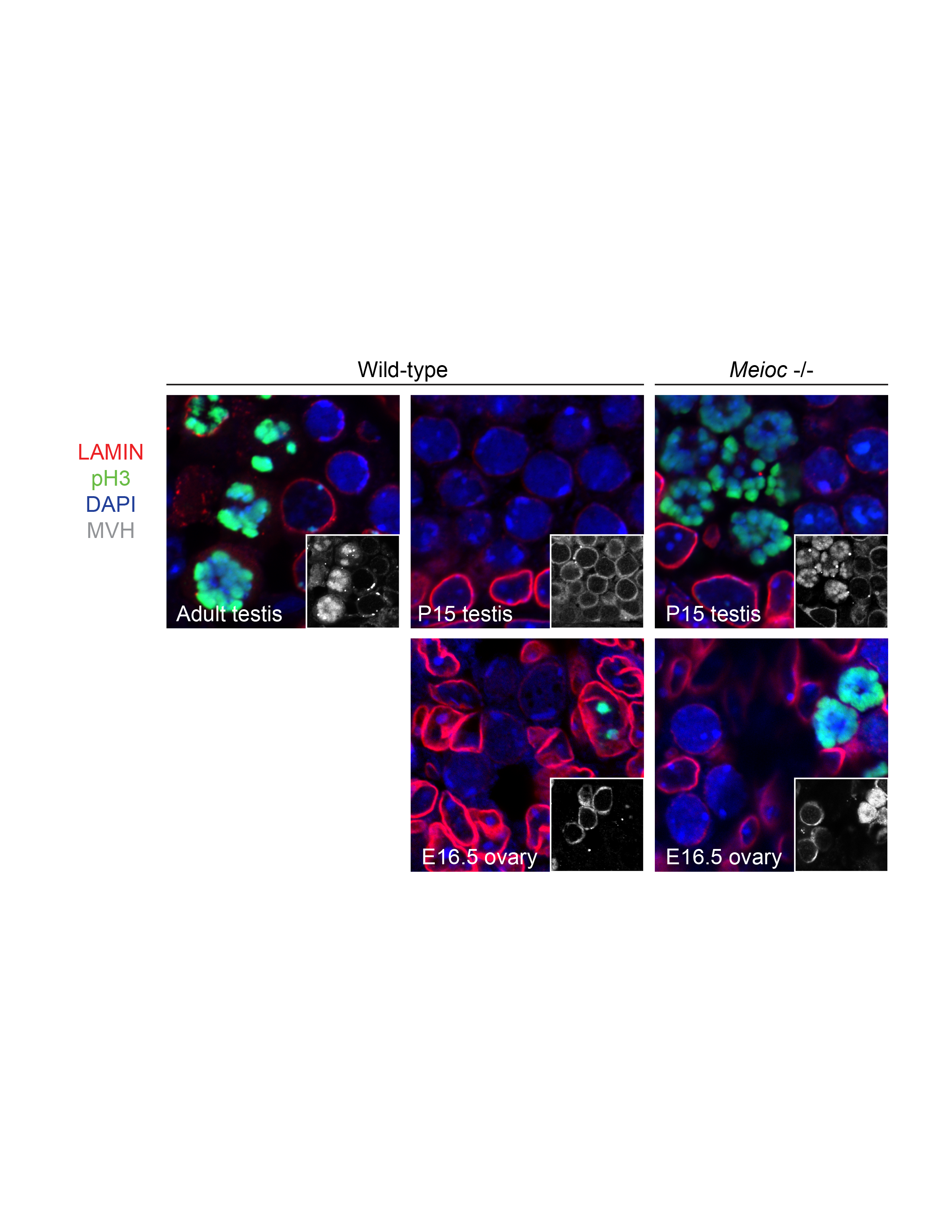

Supplement: S9 Fig — Immunofluorescence staining for LAMIN and pH3 in wild-type and Meioc-/- P15 testis and E16.5 ovary sections. Nuclei are stained by DAPI. In wild-type P15 testis and E16.5 ovary, meiotic germ cell nuclei are still intact, as detected by LAMIN staining, and no pH3 is observed. In Meioc-/- P15 testis and E16.5 ovary, LAMIN is not detected in germ cells which have condensed their nuclei and are pH3+. LAMIN and pH3 staining of wild-type adult testicular germ cells in metaphase I are shown for comparison. (TIF) [file pgen.1006704.s009.tif]

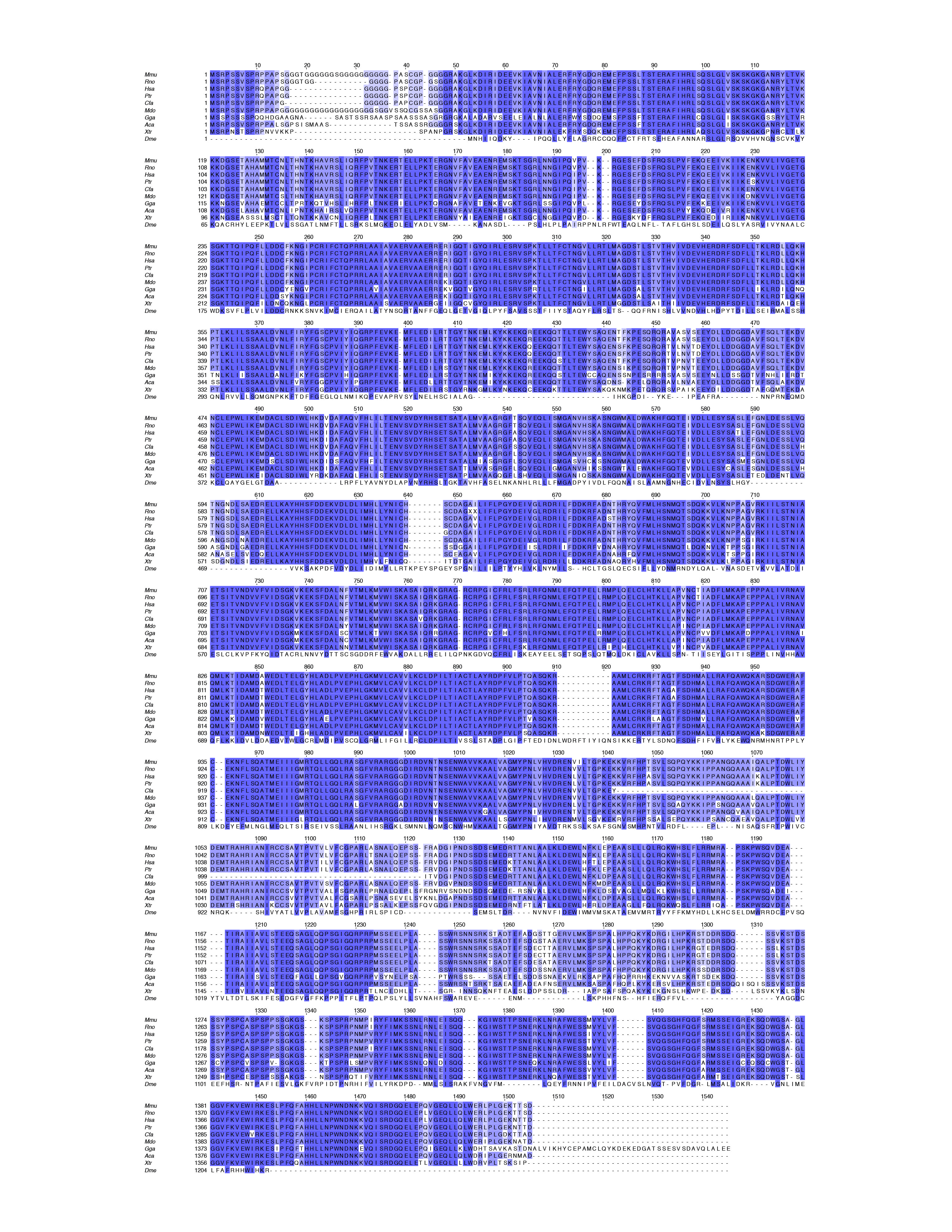

Supplement: S10 Fig — Alignment of electronic predictions of YTHDC2 orthologs. We searched for homologs of mouse YTHDC2 (NP_001156485) by querying the RefSeq protein database by blastp. We restricted the search to the following representative species: Mus musculus, Rattus norvegicus, Canis familiaris, Monodelphis domestica, Homo sapiens, Pan troglodytes, Anolis carolinensis, Gallus gallus, Xenopus tropicalis, Danio rerio, Branchiostoma floridae, Ciona intestinalis, Strongylocentrotus purpuratus, Bombyx mori, Caenorhabditis elegans, Nematostella vectensis, Petromyzon marinus, Drosophila melanogaster, Saccharomyces cerevisiae. Homologs of mouse YTHDC2 (≥75% query coverage and ≥25% identity) were aligned by Clustal Omega and visualized by Jalview (shown in figure). In Drosophila melanogaster, the homolog was annotated as benign gonial cell neoplasm (BGCN; NP_523832.2). Additional matches to YTHDC2 were found in Danio rerio, Branchiostoma floridae, Ciona intestinalis, Strongylocentrotus purpuratus, Bombyx mori, Caenorhabditis elegans, and Nematostella vectensis. We were unable to identify matches to full-length mouse YTHDC2 in Petromyzon marinus and Saccharomyces cerevisiae. (TIF) [file pgen.1006704.s010.tif]

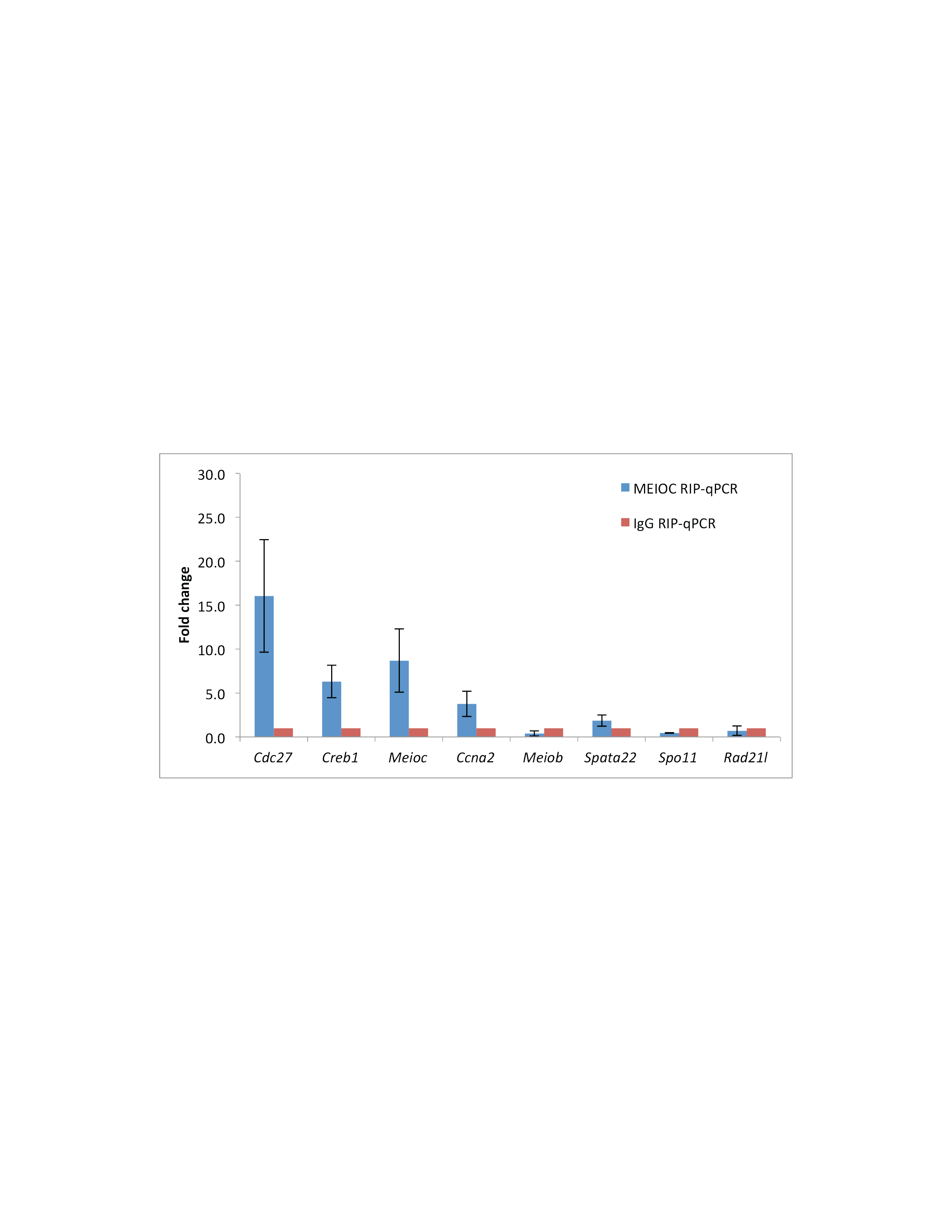

Supplement: S11 Fig — A subset of targets and non-targets, identified via RIP-Seq and enrichment analysis, were verified via qPCR of the same P15 RIP samples analyzed via sequencing (N = 2). All ΔΔCt values were normalized to Actb qPCR results and displayed as fold change over IgG RIP-qPCR. Error bars represent s.e.m. Overall trends of target abundance in MEIOC RIP compared to IgG RIP are consistent with RIP-seq results. However, statistical analysis (one-tailed, paired Student t-test) did not show the statistical enrichment of any target in the MEIOC RIP (p>0.05 for all targets). (TIF) [file pgen.1006704.s011.tif]
