# Supplementary material for: Meioc maintains an extended meiotic prophase I in mice
Source: PLoS Genet. 2017 Apr 5;13(4):e1006704. doi: 10.1371/journal.pgen.1006704 (PMC5397071; doi:10.1371/journal.pgen.1006704)
Supplement: S6 Table — (DOCX) [file pgen.1006704.s017.docx]

| **S6 Table: Antibodies and experimental conditions for immunofluorescent stainings performed in this study** | | | |
| --- | --- | --- | --- |
| **Antibody** | **Source** | **Fixation condition** | **Incubation concentration** |
| Rabbit anti-GM1564 | This study | Bouins 2 hours, paraffin embedded | 1:300 |
|  |  | PFA 1 hour, frozen in OCT | 1:300 |
| Rabbit anti-STRA8 | Abcam  ab49405 | PFA o/n, paraffin embedded | 1:100-250 |
| Goat anti-MVH | Sigma  AF2030 | PFA o/n, paraffin embedded | 1:500 |
| Rabbit anti-DMC1 | Santa Cruz Biotechnologies  sc22768 | PFA o/n, paraffin embedded | 1:200 |
|  |  | Spreads | 1:200 |
| Mouse anti-SYCP3 | Santa Cruz Biotechnologies  sc74569 | PFA o/n, paraffin embedded | 1:100-200 |
|  |  | Spreads | 1:300 |
| Rabbit anti-CENPA | Abcam  ab33565 | PFA o/n, paraffin embedded | 1:500 |
| Rat anti-aTUB | Abcam  ab6160 | PFA o/n, paraffin embedded | 1:1000 |
| Rabbit anti-LAMIN | Abcam  ab16048 | PFA o/n, paraffin embedded | 1:1000 |
| Mouse anti-pH3 | Abcam  ab14955 | PFA o/n, paraffin embedded | 1:2000 |
| Rabbit anti-CCNA2 | Abcam  ab7956 | PFA o/n, paraffin embedded | 1:300 |
| Rabbit anti-REC8 | Schultz lab | Spreads | 1:200 |
| Rabbit anti-YTHDC2 | Bethyl Laboratories A303-026A | PFA o/n, paraffin embedded | 1:500 |
| Rabbit anti-SYCP1 | Novus Biologicals  NB300-229 | Spreads | 1:100 |
| Mouse anti-phospho-Histone H2A.X (Ser139), clone JBW301 | Millipore  05-636 | Spreads | 1:250 |
