# Supplementary material for: Meioc maintains an extended meiotic prophase I in mice
Source: PLoS Genet. 2017 Apr 5;13(4):e1006704. doi: 10.1371/journal.pgen.1006704 (PMC5397071; doi:10.1371/journal.pgen.1006704)
Supplement: S8 Table — (DOCX) [file pgen.1006704.s019.docx]

| Gene | Forward primer (5’ to 3’) | Reverse primer (5’ to 3’) |
| --- | --- | --- |
| *Actb* | AGAAGGACTCCTATGTGGGTGA | CATGATCTGGGTCATCTTTTCA |
| *Creb1* | GCGGAGGTGTAGTTTGACGC | TAGTTACCGGTGGTACAAGCTCC |
| *Ccna2* | TCGCTGCATCAGGAAGACCA | AGGAGCAACCCGTCGAGTC |
| *Cdc27* | CCCCAAGTTTTGGGATTTTGCCA | TTTGTAGGTGCTCCGGTGGG |
| *Meiob* | GGGCCCGGAAGTATCCGATTTA | TCACTGCCCCAGGAGGATAC |
| *Meioc* | CTGAGGGAGGGACCTGAGCC | ATCATAGAAGGGCGCCGAGC |
| *Pum2* | CAACAGCAGCTCTTTCAGAGGACTA | AGCAAGGCCTGCTGAGAATACAC |
| *Rad21l* | GTTCTGGACCTTGCACCCCC | TGAGGGTTCTGCAATCTGCTGG |
| *Spata22* | TGCTGAAGGAGGCGAGGTTTA | AACAATGGCACAGGCAAACAGC |
| *Spo11* | CTGATCACAGATGCGAAGTTTCTG | TGCCGTAGGGATCTGCATCG |

**S8 Table. List of qPCR primers used in this study**
