# Supplementary material for: Meioc maintains an extended meiotic prophase I in mice
Source: PLoS Genet. 2017 Apr 5;13(4):e1006704. doi: 10.1371/journal.pgen.1006704 (PMC5397071; doi:10.1371/journal.pgen.1006704)
Supplement: S9 Table — (DOCX) [file pgen.1006704.s020.docx]

| **S9B Table. Coding of sample types for DESeq analysis of MEIOC RIP** | | | | |
| --- | --- | --- | --- | --- |
|  | genotype | MEIOC.specific | MEIOC.nonspecific | IgG |
| WT MEIOC RIP-seq | 1 | 1 | 1 | 1 |
| KO MEIOC RIP-seq | 0 | 0 | 1 | 1 |
| WT IgG RIP-seq | 1 | 0 | 0 | 1 |
| WT RNA-seq | 1 | 0 | 0 | 0 |
| KO RNA-seq | 0 | 0 | 0 | 0 |

| **S9A Table. Coding of sample types for DESeq analysis of YTHDC2 RIP** | | |
| --- | --- | --- |
|  | YTHDC2 | IgG |
| WT YTHDC2 RIP-seq | 1 | 1 |
| WT IgG RIP-seq | 0 | 1 |
| WT RNA-seq | 0 | 0 |
